# Tumor infiltrating T cell states and checkpoint inhibitor expression in hepatic and pancreatic malignancies

**DOI:** 10.3389/fimmu.2023.1067352

**Published:** 2023-01-31

**Authors:** Shanshan Wan, Ende Zhao, Daniel Freeman, Daniel Weissinger, Benjamin A. Krantz, Gregor Werba, Lauren G. Khanna, Despina Siolas, Paul E. Oberstein, Pratip K. Chattopadhyay, Diane M. Simeone, Theodore H. Welling

**Affiliations:** ^1^ Department of Surgery, NYU Langone Health, New York, NY, United States; ^2^ Perlmutter Cancer Center, NYU Langone Health, New York, NY, United States; ^3^ Pathology, NYU Langone Health, New York, NY, United States; ^4^ Internal Medicine, NYU Langone Health, New York, NY, United States; ^5^ Talon Biomarkers, Mendham, NJ, United States

**Keywords:** pancreatic adenocarcinoma, hepatocellular carcinoma, cholangiocarcinoma, immune oncology, tumor immunity, T cell biology, checkpoint inhibitors

## Abstract

Hepato-pancreatico-biliary (HPB) malignancies are difficult-to-treat and continue to to have a high mortality and significant therapeutic resistance to standard therapies. Immune oncology (IO) therapies have demonstrated efficacy in several solid malignancies when combined with chemotherapy, whereas response rates in pancreatic ductal adenocarcinoma (PDA) are poor. While promising in hepatocellular carcinoma (HCC) and cholangiocarcinoma (CCA), there remains an unmet need to fully leverage IO therapies to treat HPB tumors. We therefore defined T cell phenotypic states, particularly in terms of immune checkpoint receptor expression, in the tumor microenvironment of HPB patients utilizing novel, multiparameter flow cytometry and bioinformatics analysis. We demonstrate the presence of CD103^+^ tissue resident memory T cells (T_RM_), CCR7^+^ central memory T cells, and CD57^+^ terminally differentiated effector cells across all HPB cancers, with simultaneous expression of multiple co-inhibitory checkpoint receptors. Terminally differentiated T cells lacking co-stimulatory receptors were more prevalent in PDA, whereas T cells expressing both co-inhibitory and co-stimulatory receptors were most prevalent in HCC, especially in early stage. HCC patients had significantly higher TRM with a phenotype that might confer restored activation in response to immune checkpoint therapies. Further, T-cell activation state and checkpoint expression did not change robustly in response to chemotherapy in PDA patients. These results support that HCC patients might benefit most from combined checkpoint therapies, whereas efforts other than cytotoxic chemotherapy will likely be necessary to increase overall T cell activation in CCA and PDA for future clinical development.

## Introduction

Immune oncology (IO) treatments have demonstrated significant efficacy for many solid malignancies and new indications are actively under study in many ongoing clinical trials across cancer types (1, 2). Checkpoint inhibitor (CI) IO treatment has thus far been the cornerstone of these treatments, mainly targeting programmed death receptor 1 (PD-1; CD279) and its ligand PD-L1 (CD274; B7-H1) or cytotoxic T lymphocyte-associated protein 4 (CTLA4; CD152) (3). However, of hepato-pancreatico-biliary (HPB) cancers, pancreatic ductal adenocarcinoma (PDA) and cholangiocarcinoma (CCA) have been refractory to many chemotherapy regimens, including IO treatments, with the exception of hepatocellular carcinoma (HCC). While efficacy for HCC has been notable, therapeutic responses have still been limited with the initial Checkmate-40 trial evaluating nivolumab demonstrating an objective response rate (ORR) of 14% (4), and the phase III trial failing to meet statistical significance with respect to improving overall survival (OS). However, the recent ImBrave trial for advanced HCC patients evaluating atezolizumab and bevacizumab demonstrated an ORR of 27% with an improvement in median overall survival (OS) (HR 0.59, median OS 19.2 months versus 13.4 months) (5). Recently the TOPAZ-1 trial, targeting PD-L1 in addition to standard chemotherapy, demonstrated an increase in overall survival of 20% and ORR of 26.7% for advanced biliary cancer patients (2). IO therapy has thus far shown no clinical benefit in PDA patients except in cases of microsatellite instability. While many novel CI targeting agents and costimulatory checkpoint agonists are in early phase clinical trials in other cancer types, the possible efficacy and biologic rationale of these agents in HPB cancers remains to be determined.

In order to fully leverage IO treatments in HPB cancers, the tumor immune microenvironment (TME) landscape needs to be further defined to identify and evaluate immune targets as strategies for future clinical trials. Thus far the translational assessment of the HPB TME has been limited, due to insufficient tissue availability, inadequate technologies, or a lack of correlation to important clinical parameters. Consequently, the landscape and quantitation of T cell phenotypes in relation to stage, treatment, and type of HPB malignancy requires definition. Further, identifying T cell phenotypes in hepatic and pancreatic malignancies may elucidate resistance mechanisms to anti-PD-1 therapy and help to select patients for the ideal combination of IO targeting agents.

Tissue-resident memory T cells (T_RM_) are a subset of T cells with both effector and memory function, residing in peripheral tissues (6). T_RM_ cells are characterized by the expression of tissue retention markers CD103 and CD69, and reduced expression of migration potential markers, such as CCR7 (7, 8). Additionally, C-X-C motif chemokine receptor 6 (CXCR6; CD186) and PD-1 were also reported to be core markers of T_RM_ cells (9). In recent studies, T_RM_ cells were reported to be a component of tumor-infiltrating lymphocytes (TILs), associated with improved response to immunotherapy and favorable clinical outcome (6). The phenotype, functional state and implication of T_RM_ cells in HPB cancers is still unclear and needs further investigation.

In this study we analyzed tumors from 36 patients with HPB malignancies, including PDA, (HCC), and intrahepatic CCA. We hypothesized that distinct T cell phenotypes are present in each cancer type and that T cell phenotypes may correlate with clinical stage and prognosis. To explore these hypotheses, we utilized a novel high parameter flow cytometry platform to identify T cell activation states, and checkpoint molecule expression based on T cell surface marker expression. Our data show distinct HPB tumor microenvironmental T cell states and is the first study demonstrating major differences in T cell phenotypes and checkpoint molecule surface expression among HCC, PDA and CCA patients.

## Methods

### Patient subjects, tissue procurement and processing

Thirty-six patients with PDA (n=20), HCC (n=11), or intrahepatic CCA (n=5), identified at our multidisciplinary pancreatic and liver tumor programs underwent informed consent for tissue procurement and clinical-pathological demographic information under an IRB approved protocol. All samples were confirmed to be malignant by pathologist review. Subjects who were being considered for surgical resection were eligible as were PDA patients undergoing biopsy in preparation for either standard of care neoadjuvant chemotherapy or for chemotherapy as primary treatment. Biopsies of PDA patients were performed of either primary pancreatic tumors or liver metastases by EUS or percutaneous radiographic guidance, respectively (2-4 18-gauge cores). All HCC specimens were collected from surgical resection. CCA specimens were collected from either biopsy (n=2) or surgical resection (n=3). Freshly procured tissue was immediately placed in tissue storage media at 4°C, which was processed into small pieces with a scalpel and enzymatically digested into single cell suspensions in gentleMACS C tubes with Human Tissue Dissociation Kit by gentleMACS Octo Dissociator with Heaters (Miltenyi Biotec) for 30 minutes, then filtered through a 40 uM strainer. Cells were pelleted and freshly stained with antibodies for flow cytometry analysis within two hours from time of sample acquisition.

### High parameter flow cytometry

A 25 color antibody panel was developed using ColorWheel software (10) to include T cell markers of differentiation, activation and trafficking along with co-inhibitory and co-stimulatory checkpoint molecules. The following mouse anti-human antibodies were used, all purchased from BD Biosciences, except where indicated: CD186 (Brilliant Blue (BB)515), CD137 (BB630), CD244 (BB700), CD57 (BB780), CD45RO (allophycocyanin (APC)), HLA-DR (R700APC), GITR (Cyanin 7 (Cy7)-APC), CD278 (Brilliant Violet (BV)421)), CD95 (BV510), CD103 (BV605), CD183 (BV650), CD134 (BV705), CD69 (BV750), CD4 (BV785), CCR7 (Brilliant Ultraviolet (BUV) 395), LIVE/DEAD Fixable Blue (ThermoFisher Scientific, Carlsbad, CA), CD3 (BUV496), CD25 (BUV563), CD366 (BUV661), PD-1 (BUV737), CD8 (BUV805), TIGIT (phycoerythrin (PE)), CD272 (PE-CF594), CD127 (PE-Cy5) and CD152 (PE-Cy7) (10). Data were collected on a custom BD Biosciences FACSymphony A5 30-parameter flow cytometer, then compensated and analyzed with FlowJo software (BD Biosciences). Data were checked for quality of staining and fluorescence aggregates, dead cells and cell doublets were excluded. Fluorescence intensity thresholds were then determined for each marker to distinguish positive from negative expression.

### Dimension reduction analysis

Unbiased identification of cell clusters was performed using FlowJo software with Flow cytometry-based self-organizing maps (FlowSOM) (11) and t-distributed stochastic neighbor embedding (tSNE) (12) analysis. We used a combination of unbiased dimension reduction based on marker expression and manual gating validation of distinct cell clusters to analyze our datasets and compare among clinical parameters. The cell number of each sample was adjusted to a similar number by DownSample plugin in FlowJo and concatenated into one file to keep comparable cell numbers among different samples. The data was analyzed with tSNE, FlowSOM and ClusterExplorer plugins in FlowJo downloaded from https://www.flowjo.com/exchange/.

### Combinatorial analysis

CD4^+^ and CD8^+^ T cell data were exported respectively for each sample for rapid computation of combinatorial phenotypes using the CytoBrute platform (RocketML) (10). Phenotypes consisting of every single marker, and all possible combinations of 2-15 markers were constructed, and enumerated across all samples. The top 1,000 most frequent combinatorial phenotypes were reported and compared across study groups. For some analyses, flow cytometry data files, gating thresholds and comparison groups were uploaded to TerraFlow (TerraFlow.app) for construction of all possible 1-5 marker phenotypes across the dataset. Phenotypes whose frequency differed statistically significantly between phenotypes were reported. TerraFlow also generated core phenotypes that summarize the most significant families of phenotypes. Finally, a simple set of markers that distinguished groups was identified using recursive feature elimination (13).

### Statistical analysis

Data were presented in boxplot as median ± interquartile range (IQR): box middle lines, median; box limits upper and lower quartiles; box whiskers, 1.5x the interquartile range. Group comparisons were performed with the Mann-Whitney U test. Multiple comparisons were performed with the Kruskal-Wallis test. P < 0.05 were considered statistically significant.

## Results

### Patient clinical presentation and sample analysis

Tumor tissue from HCC (n=11 patients), CCA (n=5 patients) and PDA (n=20 patients) were collected from HPB cancer patients by either core needle biopsy or at time of surgical resection. The stage and clinical characteristics of these patients are listed in **Table 1**. HCC and intrahepatic CCA patients frequently had underlying liver disease (72 and 60%, respectively) with NASH, HBV, and HCV being the most common etiologies at 9, 27, and 36%, in HCC patients. None of the HCC patients had prior therapy. One of the CCA patients (20%) received chemotherapy (Gemcitabine + Cisplatin) before biopsy. Of the PDA patients, 45% had received prior neoadjuvant chemotherapy consisting of gemcitabine-based (20%) or FOLFIRINOX (25%) chemotherapy. Two PDA patients (10%) had received radiotherapy along with their chemotherapy. No PDA stage IV patients (n=5) had received chemotherapy prior to biopsy. PDA primary patient tumors demonstrated advanced disease of either lymph node metastasis or distant metastasis in 65% of patients. 35% (n=7) of PDA cases were from core biopsy tissue (2-4 18-gauge cores). Samples were accepted for analysis if there was sufficient CD4^+^ and CD8^+^ content (n>200 cells), respectively.

**Table 1:** Patient clinical characteristics and demographics.

Variable	HCC (n=11)	CCA (n=5)	PDA (n=20)
Gender, male (%)	11 (100)	0 (0)	8 (40)
Median age (range)	65 (40-84)	75 (69-78)	70 (38-85)
Etiology			
HBV (%)	3 (27)	0	NA
HCV (%)	4 (36)	0	NA
NASH (%)	1 (9)	3 (60)	NA
Stage			
I (%)	7 (64)	1 (20)	4 (20)
II (%)	1 (9)	2 (40)	8 (40)
III (%)	3 (27)	2 (40)	3 (15)
IV (%)	0	0	5 (25)
Vascular invasion (%)	4 (36)	0	0
Lymph node involvement (%)	0	3 (60)	9 (69)*
Metastasis (%)	0	0	4 (20)
Neoadjuvant (%)	0	1 (20)	7 (35)
Gemcitabine-based (%)	0	1 (20)	4 (20)
FOLFIRINOX-based (%)	0	0	3 (15)
Radiation (%)	0	0	2 (10)
Tissue type			
Biopsy (%)	0 (0)	2 (40)	7 (35)
Resection (%)	11 (100)	3 (60)	13 (65)
Mutation (%)**			12 (60)
KRAS (%)	NA	NA	11 (92)
TP53 (%)	NA	NA	9 (75)
P16 (%)	NA	NA	6 (50)
SMAD4 (%)	NA	NA	3 (25)

* LN metastasis in resection tissue only.

** In sequenced tissue only.

### T cell distribution in HPB cancers

**Figure 1:**
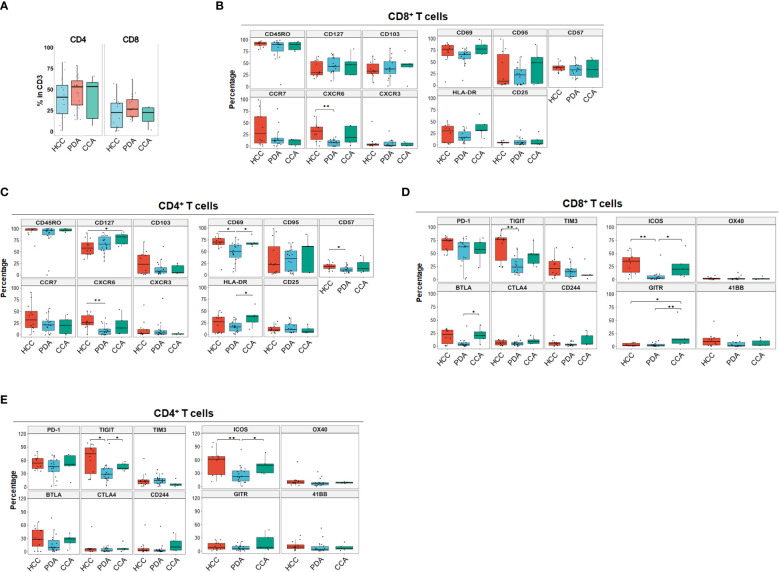
Landscape of T cell differentiation, activation state, inhibitory and stimulatory checkpoint receptors of tumor infiltrating T cells in HCC, PDA and CCA. **(A)** Percentage of CD4+ T cells and CD8+ T cells in CD3+ T cells in HCC (n=11), PDA (n=20) and CCA (n=5) patients. **(B, C)** Boxplots showing frequency of T cell differentiation markers and activation markers in CD8+ T cells **(B)** and CD4+ T cells **(C)** in HCC, PDA and CCA patients. Box middle lines, median; box limits upper and lower quartiles; box whiskers, 1.5x the interquartile range. Kruskal-Wallis test. **(D)** Inhibitory and stimulatory checkpoint receptors frequency of CD8+ T cell. Box middle lines, median; box limits upper and lower quartiles; box whiskers, 1.5x the interquartile range. *p < 0.05; ** p < 0.01; Kruskal-Wallis test. **(E)** Inhibitory and stimulatory checkpoint receptors frequency of CD4+ T cell. Box middle lines, median; box limits upper and lower quartiles; box whiskers, 1.5x the interquartile range. *p < 0.05; ** p < 0.01; Kruskal-Wallis test.

We began by quantitating the distribution of T cell subsets in the 3 HPB cancer types, to identify whether there may be distinct features in CD4^+^ and CD8^+^ T cell differentiation, activation, trafficking, phenotype, co-inhibitory and co-stimulatory checkpoint molecules in the tumor microenvironment (TME) using single marker analysis. The overall percentage of T cells were similar among all three cancer types. The majority of TILs were CD4^+^ T cells in all HPB tumors (**Figure 1A**), with an average percentage of 43.9% (1.15-82.7). PDA, HCC and CCA patients had a similar percentage of CD4^+^ T cells, CD8^+^ T cells, and CD4/CD8 ratio. In PDA, the median percentage of CD4^+^ T cells and CD8^+^ T cells were 54.6% (14.2%-78.6%) and 25.8% (12.2%-62.1%), and the CD4/CD8 ratio was 2 (0.23-4.42). In HCC, the median percentage of CD4^+^ T cells and CD8^+^ T cells were 40.95% (1.15%-82.6%) and 22.7% (0.35%-56.9%), and the CD4/CD8 ratio was 1.7 (0.56-71.29). In CCA, the median percentage of CD4^+^ T cells and CD8^+^ T cells were 53.4% (7.37%-66%) and 22.7% (1.98%-29.5%), and the CD4/CD8 ratio was 2.32 (1.25-3.72) (**Figure 1A**). The overwhelming majority (>90%) of CD4^+^ and CD8^+^ TILs in both PDA and HCC were antigen-experienced memory T cells (CD45RO^+^) (**Figures 1B, C**). Here we assessed tissue resident memory T cells (T_RM_), central memory (T_CM_) and terminally differentiated effector T cells. CD103, a marker for T_RM_ cells with increased tumor antigen sensitivity and improved response to immunotherapy, was more prevalent in CD8^+^ than CD4^+^ cells in overall HPB patients. T_RM_-associated tissue homing markers CD69 and CXCR6 were highest in HCC TILs (**Figures 1B, C**) (14, 15) suggesting superior T_RM_ recruitment and retention in HCC compared to PDA and CCA. Based on CCR7 expression (16), a marker for T_CM_ with migratory capacity to secondary lymphoid organs (SLO) (17), a median of 27.37% (0.53%-99.22%) of CD8^+^ T cells and 31.98% (0-90.96%) of CD4^+^ T cells in HCC had a T_CM_ phenotype; In PDA, 12.61% (0.26%-80.13%) of CD8^+^ T cells and 21.32% (0-56.97%) of CD4^+^ T cells had a T_CM_ phenotype; In CCA, 13.17% (0.68%-14.85%) of CD8^+^ T cells and 20.29% (0.22-43.58%) of CD4^+^ T cells were T_CM_. T_CM_ in HCC trended higher when compared to T_CM_ in PDA and CCA, but did not reach statistical significance, based on CCR7 as a single marker (**Figures 1B, C**). CD127 (the IL7-receptor) also commonly marks TCM, but exhibited a different pattern than CCR7, as it trended to higher levels (amongst CD8^+^ T-cells) or is significantly elevated (amongst CD4^+^ T-cells) in PDA and CCA compared to HCC. CD57 is a marker for terminally differentiated effector T cells (18, 19). CD57+ memory CD8+ T effector cells have been shown to express cytolytic enzymes such as perforin and granzymes but may be more susceptible to apoptosis and senescence following stimulation (20). CD57^+^CD4^+^ T cells were 17.82% (6.68%-32.1%), 10.48% (3.98%-25.9%), 13.28% (5.26%-41.1%) in HCC, PDA and CCA, respectively, and CD57^+^ CD8^+^ T cells were 37.81% (24.83%-56.21%), 33.03% (11.67%-60.35%), 18.17% (16.83%-58.52%) in HCC, PDA and CCA respectively (**Figures 1B, C**). These findings demonstrated the presence of T_RM_, T_CM_ and terminally differentiated effector CD8^+^ and CD4^+^ TILs with similar frequencies among HPB patients.

### Immune checkpoint expression in HPB malignancies

Immune checkpoint molecules play crucial regulatory roles in promoting or suppressing anti-tumor immune responses and are therefore a target of ongoing therapeutic development for cancer immune therapy (21). The most common co-stimulatory and co-inhibitory immune checkpoint receptors on T cells were analyzed for each individual marker distribution in HPB patients (**Figures 1D, E**). Overall, co-inhibitory immune checkpoint receptors were more frequently expressed than co-stimulatory immune checkpoint receptors in HPB patients with PD-1 and TIGIT being the most frequent (**Figures 1D, E**). PD-1^+^ percentage in CD8^+^ T cells in HCC were 74.76% (46.79%-84%) compared to PDA 62.96% (1.52%-82.83%) and CCA 56.4% (22.98%-79.77%), although not statistically significant. PD-1^+^ percentage in CD4^+^ T cells were comparable among HCC 53.9% (35.77%-80.03%), PDA 45.76% (3.67%-72.11%) and CCA 51.15% (9.66%-71.16%). Notably, in all of the HCC patients of our cohort, PD-1^+^ percentage exceeded 46% and 35% in CD8^+^ and CD4^+^ T cells. In contrast, both PDA and CCA patients showed a wider range of PD-1 expression. In addition, TIGIT was expressed at a significantly higher frequency in HCC and CCA patients in CD4^+^ and in HCC CD8^+^ T cells compared to PDA patients. BTLA was expressed significantly higher in CD8^+^ T cells of CCA samples. This data suggests that HCC samples had more antigen-experienced T cells expressing co-inhibitory checkpoints such as PD-1, TIGIT and BTLA. Notably, CTLA4 was expressed at the lowest frequency of co-inhibitory immune checkpoint receptors in overall HPB patients (<9% median percentage, p < 0.001 compared to PD-1) (**Figures 1D, E**). Co-stimulatory checkpoint receptors median percentage of expression was less than 12% for OX40 (CD134), GITR, and 4-1BB (CD137) (**Figures 1D, E**). ICOS was the highest expressed co-stimulatory immune checkpoint receptor and significantly higher in HCC (35.38% in CD8, 61.66% in CD4), and CCA (23.27% in CD8, 48.19% in CD4), compared to PDA patients (3.27% in CD8, 22.34% in CD4). GITR was significantly higher in CD8^+^ T cells from CCA patients, compared to CD8^+^ T cells from HCC or PDA patients. These data demonstrated distinct single marker immune checkpoint expression profiles among HCC, PDA and CCA.

### T cell phenotypes unique to HPB cancer type

**Figure 2:**
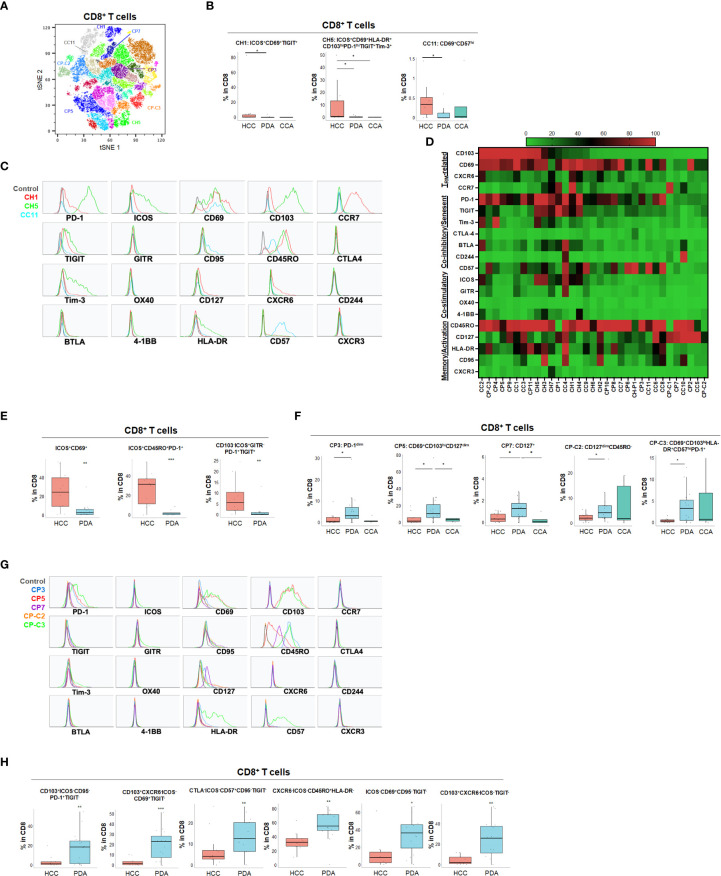
Distinct CD8+ T cell phenotypes in liver and pancreatic cancer. **(A)** t-distributed stochastic neighbor embedding (tSNE) projection of CD8+ T cells showing manual-gated clusters identified from HCC (n=11), PDA (n=20) and CCA (n=5) patients. Significantly enriched clusters in HCC (CH1, CH5, CC11) and PDA (CP3, CP5, CP7, CP-C2, CP-C3) are labeled. **(B)** Boxplots showing percentage of indicated HCC-enriched clusters and phenotype for CH1, CH5 and CC11 in CD8+ T cells from tSNE (A) comparing HCC, PDA and CCA. Box middle lines, median; box limits upper and lower quartiles; box whiskers, 1.5x the interquartile range. *p < 0.05; Kruskal-Wallis test. **(C)** Histograms showing individual marker expression of the HCC-enriched clusters CH1 (red), CH5 (green) and CC11 (blue) in CD8+ T cells. **(D)** Expression of TRM-related markers, co-inhibitory/senescent markers, co-stimulatory markers and memory/activation markers in 33 clusters of CD8+ T cells is shown in heatmap which is arranged based on the expression level of CD103. **(E)** Combinatoric frequency analysis of CD8+ T cell marker combinations (TerraFlow-Methods) most frequent in HCC. Boxplots showing representative phenotypes of CD8+ T cell that are significantly increased in HCC compared to PDA. Box middle lines, median; box limits upper and lower quartiles; box whiskers, 1.5x the interquartile range. **p < 0.01; *** p < 0.001; Kruskal-Wallis test. **(F)** Boxplots showing percentage of indicated PDA-enriched clusters and phenotype for CP3, CP5, CP7, CP-C2 and CP-C3 in CD8+ T cells from tSNE (A). Box middle lines, median; box limits upper and lower quartiles; box whiskers, 1.5x the interquartile range. *p < 0.05, Kruskal-Wallis test. **(G)** Histograms of individual marker expression of the PDA-enriched clusters CP3 (blue), CP5 (red), CP7 (purple), CP-C2 (orange) and CP-C3 (green) in CD8+ T cells. **(H)** Combinatoric frequency analysis of CD8+ marker combinations (TerraFlow-Methods) most frequent in PDA. Boxplot showing phenotypes of CD8+ T cell that are significantly higher in PDA compared to HCC. Representative statistically and biologically significant phenotypes are shown. Non-parametric Mann-Whitney U test *p<0.05, **p<0.01, ***p<0.001.

**Figure 3:**
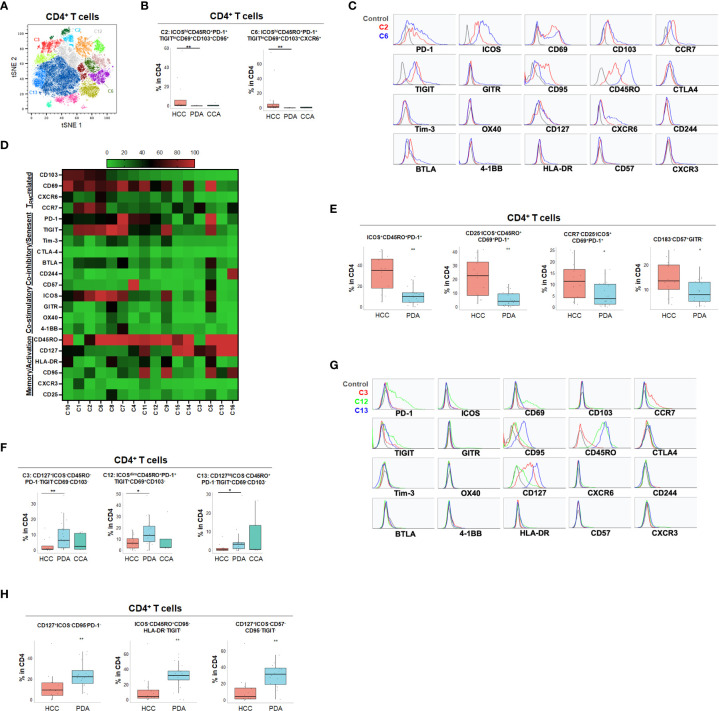
Distinct CD4+ T cell phenotypes in liver and pancreatic cancers. **(A)** tSNE projection of CD4+ T cells showing 16 manual-gated clusters identified from HCC (n=11), PDA (n=20) and CCA (n=5) patients. Significantly enriched clusters in HCC (C2, C6) and PDA (C3, C12, C13) are labeled. **(B)** Boxplots showing percentage of HCC-enriched clusters C2 and C6 in CD4+ T cells from tSNE (A). Box middle lines, median; box limits upper and lower quartiles; box whiskers, 1.5x the interquartile range. **p < 0.01, Kruskal-Wallis test. **(C)** Histograms show the individual marker expression of CD4+ T cell clusters enriched in HCC, C2 (red) and C6 (blue). **(D)** Expression of TRM-related markers, co-inhibitory/senescent markers, co-stimulatory markers and memory/activation markers in 16 clusters of CD4+ T cells is shown in heatmap which is arranged based on the expression level of CD103. **(E)** Combinatoric frequency analysis of CD4+ marker combinations (TerraFlow-Methods) most frequent in HCC. Boxplots showing representative phenotypes of CD4+ T cells that are significantly higher in HCC compared to PDA. Box middle lines, median; box limits upper and lower quartiles; box whiskers, 1.5x the interquartile range. *p < 0.05, **p < 0.01, Kruskal-Wallis test. **(F)** Boxplots showing percentage of PDA-enriched clusters C3, C12 and C13 in CD4+ T cells from tSNE (A). Box middle lines, median; box limits upper and lower quartiles; box whiskers, 1.5x the interquartile range. *p < 0.05,**p < 0.01, Kruskal-Wallis test. **(G)** Histograms showing the individual marker expression CD4+ T cell clusters enriched in PDA of C3 (red), C12 (green) and C13 (blue). **(H)** Combinatoric frequency analysis of CD4+ marker combinations (TerraFlow-Methods) most frequent in PDA. Boxplots showing representative phenotypes of CD4+ T cell that are significantly higher in PDA compared to HCC. Representative statistically and biologically significant phenotypes are shown. Box middle lines, median; box limits upper and lower quartiles; box whiskers, 1.5x the interquartile range. **p < 0.01, Kruskal-Wallis test.

Using specialized data analysis tools, like CytoBrute and TerraFlow, high parameter flow cytometry data can be mined to examine all the theoretically possible phenotypes. In this study, as many as 3^22^ theoretical phenotypes may be present in the data (where three conditions – positive, negative, and omitted are analyzed for each of 22 markers). Because some markers were rarely expressed, and because of the challenge of interpreting long higher-order phenotypes of marker combinations, we limited CytoBrute analysis to 15 markers and TerraFlow analysis was designed to sample all possible sets of six markers. Cell phenotypes that were exceedingly rare were removed from downstream analysis and comparison across patient groups. While the single marker analysis noted some differences among HPB cancers, we set out to identify unique, multi-parameter, T cell populations in these 3 distinct tumor types and ultimately to identify T cell populations of potential clinical significance. We first utilized unbiased hierarchical clustering and t-distributed stochastic neighbor embedding (tSNE) plots along with supervised annotation to visualize T cell subsets and significant phenotypes among HPB cancer patients (**Figure 2A**). tSNE projection of CD8^+^ T cells identified 33 clusters in HCC (n=11), PDA (n=20) and CCA (n=5) patients. Statistically significant enriched clusters in HCC (CH1, CH5, CC11) and PDA (CP3, CP5, CP7, CP-C2, CP-C3) were identified. When examining the CD8^+^ T cell compartment, several populations were noted in HCC patients to be more prevalent than in PDA and CCA patients (**Figure 2B** and **Supplementary Figure 1**). HCC patients demonstrated higher CD103^+^CD69^+^ T_RM_ cells expressing co-stimulatory immune checkpoint receptor ICOS. Notably, these HCC-enriched ICOS^+^ T_RM_ frequently also expressed one or more co-inhibitory immune checkpoint receptors including PD-1, TIGIT and Tim-3 (**Figures 2C, D** and **Supplementary Figures 1C, D**). These T cells are consistent with the phenotype of T partial exhausted (T_PEX_) which are exhausted memory cells that are capable of generating anti-tumor T effector (T_eff_) cells in response to immune checkpoint blockade. Distinct HCC-enriched clusters shown in tSNE, CH1 (ICOS^+^CD69^+^TIGIT^+^) and CH5 (ICOS^+^CD69^+^CD103^hi^ HLA-DR^+^PD-1^hi^TIGIT^+^Tim-3^+^) demonstrated that ICOS^+^ T_RM_ cells could potentially be suppressed by multiple co-inhibitory checkpoint receptors in HCC (**Figures 2B, D**). ICOS^+^ clusters CH1 and CH5 were significantly lower in PDA. Similarly, CH5 was significantly lower in CCA (**Figure 3B**). We used TerraFlow to provide further insight into the results observed in our other analyses. TerraFlow identified a number of phenotypes, not revealed by tSNE analysis, that differed between HCC and PDA (**Figure 2E**), variously highlighting the elevation of ICOS, TIGIT, CD69, and PD1 in distinguishing PDA from HCC (**Supplementary Figures 2A–E**) using machine learning and recursive feature analysis (14). TerraFlow revealed that HCC was highly enriched for ICOS+ TRM that frequently expressed PD-1 and/or TIGIT (**Figure 2E**), suggesting that immune checkpoint blockade of PD1 and/or TIGIT might release inhibition of the already expressed costimulatory molecule ICOS. **Supplemental Figures 2C, F** illustrate the p-values for all phenotypes compared between HCC and PDA. Importantly, the two critical markers that distinguished HCC and PDA-infiltrating CD8^+^ T cells were ICOS and CD69 (**Supplementary Figures 2A–C**). HCC was highly enriched for ICOS^+^ T_RM_ that frequently expressed PD-1 and/or TIGIT (**Figure 2E**), further indicating the effector potential by boosting T_RM_ *via* immune checkpoint blockade. In addition, a discrete T cell cluster CC11 (CD69^+^CD57^hi^) was enriched in HCC, suggesting HCC patients had more terminally differentiated T cells with potent effector function but can become senescent and undergo activation-induced cell death (**Figures 2B–D**). In HCC, two CD4^+^ T cell clusters, C2 and C6, were noted to have unique CD69^+^CD103^+^ T_RM_ populations expressing ICOS, PD-1 and TIGIT (**Figures 3A–D** and **Supplementary Figures 3A–D**). C2 expressed higher CD95 and BTLA than C6, suggesting heterogeneity among these two populations with exhausted features. Consistently, TerraFlow analysis showed that HCC was enriched for CD4^+^ T cells expressing both co-stimulatory ICOS and co-inhibitory checkpoint PD-1, as well as CD57^+^ effector cells (**Figure 3E**).

In PDA, unique CD8^+^ or CD4^+^ T cells phenotypes were enriched that frequently expressed co-inhibitory immune checkpoint PD-1 but were lacking all co-stimulatory immune checkpoint receptors including ICOS, OX40, 4-1BB and GITR (**Figures 2F–H**, **3F–H** and **Supplementary Figures 3E, F**). PDA patients were enriched for PD-1^+^CD103^-^ CD8^+^ T population (CP3) and PD-1^+^CD8^+^ T_RM_ (CP5, CP-C3). PDA patients had higher naïve CD8^+^ T cells (CP-C2: CD45RO^-^) and effector memory T cells (T_EM_) (CP7: CD45RO^+^CD127^+^CCR7^-^) that were lacking ICOS and PD-1 expression. Interestingly, one subset of T_RM_ (CP-C3) also exhibited high CD57 expression in addition to PD-1, indicating exhaustion/senescence. Both HCC and CCA had significantly lower frequency of ICOS^-^ T_RM_ (CP5) and T_EM_ (CP7) compared to PDA. Consistently PDA was enriched for CD4^+^ T cells lacking co-stimulatory checkpoint receptors, including naïve CD4^+^ T cells (C3: CD127^+^CD45RO^-^PD-1^-^), and memory CD4^+^ T cells that expressed PD-1 and/or TIGIT (C12: CD69^+^CD45RO^+^PD-1^+^ TIGIT^+^ and C13: CD127^hi^CD45RO^+^ PD-1^-^TIGIT^+^) (**Figures 3F, G**). All of these PDA-enriched T cell populations shared a striking characteristic which is the absence of all four co-stimulatory immune checkpoint receptors, ICOS, GITR, OX40 and 4-1BB (**Figures 2H**, **3D–H**, **Supplemental Figure 3F**), suggesting T cell anergy. These findings suggest the tumor microenvironment in PDA could suppress activation of anti-tumor T cells and/or induce T cell anergy by repressing co-stimulatory immune checkpoint signals (22). The hyporesponsive state of these anergic T cells may contribute to tumor immune evasion and resistance to immune checkpoint blockade such as anti-PD-1/PD-L1 and anti-CTLA-4. Altogether, with tSNE and combinatorial expression analysis, we identified dysfunctional T cell populations uniquely enriched in HCC, CCA and PDA patients. HCC patient tumors showed higher T_PEX_ cells expressing co-stimulatory immune checkpoint ICOS and multiple co-inhibitory immune checkpoint receptors, most frequently PD-1 and TIGIT; whereas, PDA contained more anergic PD-1^+^ T cells lacking co-stimulatory immune checkpoint receptors, indicating PDA as a less immune responsive tumor compared with HCC and CCA.

### T cell phenotypes associated with HPB cancer clinical prognosis

**Figure 4:**
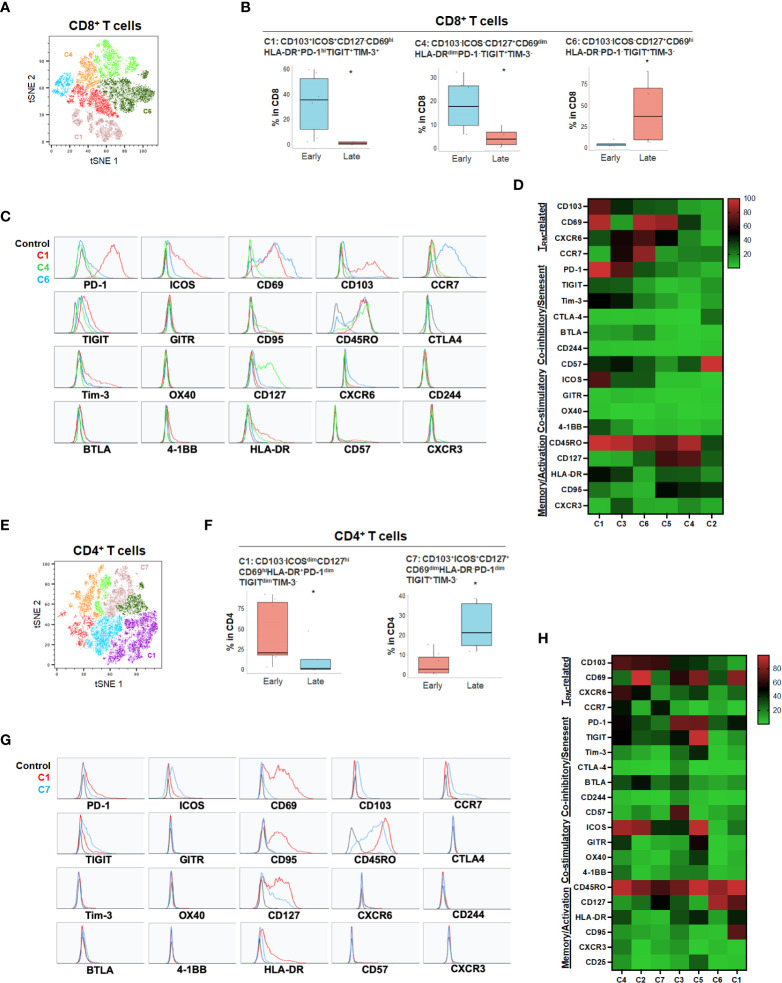
HCC disease stage and effect on T cell phenotypes. **(A)** tSNE projection of CD8+ T cells showing 6 manual-gated clusters identified from early stage (n=6) and late stage (n=4) HCC patients. Significantly enriched clusters in early stage (C1, C4) and late stage (C6) HCC patients are labeled. **(B)** Boxplots showing percentage of early stage-enriched CD8+ T cell clusters C1 and C4 and late stage-enriched cluster C6 in HCC patients. *p < 0.05, Kruskal-Wallis test. **(C)** Histograms showing the individual marker expression of early stage-enriched CD8+ T cell clusters C1 (red), C4 (green), and late stage-enriched cluster C6 (blue) in HCC patients. **(D)** Expression of TRM-related markers, co-inhibitory/senescent markers, co-stimulatory markers and memory/activation markers in 6 clusters of CD8+ T cells is shown in heatmap which is arranged based on the expression level of CD103. **(E)** tSNE projection of CD4+ T cells showing 7 manual-gated clusters identified from early stage (n=7) and late stage (n=5) HCC patients. Significantly enriched clusters in early stage (C1) and late stage (C7) HCC patients are labeled. **(F)** Boxplots showing percentage of early stage HCC enriched CD4+ T cell cluster C1 and late stage HCC enriched cluster C7. *p < 0.05, Kruskal-Wallis test. **(G)** Histograms showing individual marker expression of early stage HCC enriched CD4+ T cell cluster C1 (red) and late stage HCC enriched cluster C7 (blue). **(H)** Expression of TRM-related markers, co-inhibitory/senescent markers, co-stimulatory markers and memory/activation markers in 7 clusters of CD4+ T cells is shown in heatmap which is arranged based on the expression level of CD103.

While the tSNE analysis allowed identification of unique T cell populations in HPB cancers, we sought to further account for the heterogeneity among patients and quantify populations most likely to have clinical significance. Therefore, we evaluated whether individual T cell subpopulations were predictive of clinical prognosis in both HCC and PDA patients based on unbiased hierarchical analysis. Early stage HCC patients (Stage I-II) demonstrated a greater degree of partially exhausted CD8^+^ T_RM_ co-expressing ICOS, PD-1, TIGIT and Tim-3 (**Figure 4** and **Supplementary Figure 4**). Specifically, early stage HCC tumors showed a significant increase in CD103^+^CD8^+^ T_RM_ expressing high PD-1 and ICOS (C1) and CD127^+^ CD8^+^ and CD4^+^ T_EM_ (C4 in CD8 and C1 in CD4) when compared to late stage patients (stage IIIA-B) (**Figure 4**). Late stage HCC tumors showed more CD8^+^ (C6) and CD4^+^ (C7) memory T cells co-expressing TIGIT, a marker for exhaustion (**Figure 4**). Thus, combined blockade of PD-1, TIGIT and/or Tim-3 could be potentially beneficial in HCC patients.

**Figure 5:**
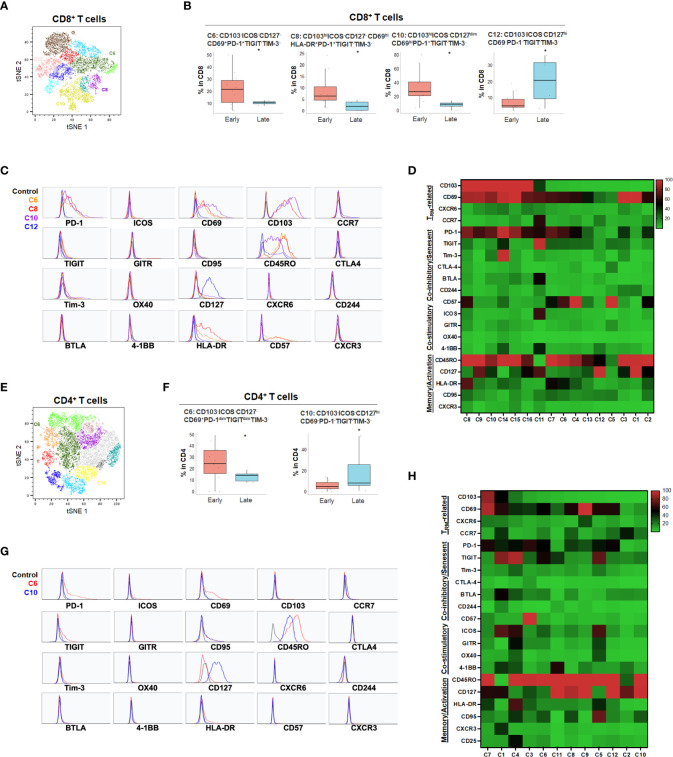
PDA disease stage and effect on T cell phenotypes **(A)** tSNE projection of CD8+ T cells showing 16 manual-gated clusters identified from early stage (n=11) and late stage (n=4) PDA patients. Significantly enriched clusters in early stage (C6, C8, C10) and late stage (C12) PDA patients are labeled. **(B)** Boxplots showing percentage of early stage-enriched CD8+ T cell clusters and late stage-enriched clusters. Box middle lines, median; box limits upper and lower quartiles; box whiskers, 1.5x the interquartile range. *p < 0.05, Kruskal-Wallis test. **(C)** Histograms showing individual marker expression of early stage-enriched CD8+ T cell clusters in C6 (orange), C8 (red), C10 (purple) and late stage-enriched cluster C12 (blue). **(D)** Expression of TRM-related markers, co-inhibitory/senescent markers, co-stimulatory markers and memory/activation markers in 16 clusters of CD8+ T cells is shown in heatmap which is arranged based on the expression level of CD103. **(E)** tSNE projection of CD4+ T cells showing 12 manual-gated clusters identified from early stage (n=13) and late stage (n=5) PDA patients. Significantly enriched clusters in early stage (C6) and late stage (C10) PDA patients are labeled. **(F)** Boxplots showing percentage of early stage-enriched CD4+ T cell cluster C6 and late stage-enriched cluster C10. Box middle lines, median; box limits upper and lower quartiles; box whiskers, 1.5x the interquartile range. *p < 0.05, Kruskal-Wallis test. **(G)** Histograms showing the individual marker expression of early stage-enriched CD4+ T cell clusters C6 (red) and late stage-enriched clusters C10 (blue). **(H)** Expression of TRM-related markers, co-inhibitory/senescent markers, co-stimulatory markers and memory/activation markers in 12 clusters of CD4+ T cells is shown in heatmap which is arranged based on the expression level of CD103.

For primary pancreatic tumors, late stage (III-IV) PDA demonstrated higher ICOS^-^PD-1^-^CD127^hi^CD8^+^ (C12) and CD4^+^ T cells (C10) compared to early stage PDA tumors (Stage I-II) (**Figure 5** and **Supplementary Figure 5**). The absence of co-stimulatory immune checkpoint receptors and PD-1 in more advanced PDA disease suggests hypo-responsive T cell phenotypes that may not be properly activated even when co-inhibitory checkpoints were blocked. Early-stage PDA patients showed increase of CD8^+^ CD103^+^ and/or CD69^+^ T_RM_ that also expressed PD-1 and/or HLA-DR (C6, C8 and C10), and higher CD4^+^ memory T cells with low level of PD-1 (C6) (**Figure 5**), suggesting that early-stage PDA contained more activated T_RM_ expressing PD-1. However, there was a noted absence of co-stimulatory checkpoint receptors, possibly limiting their full activation (**Figures 5C, D, G, H**). Additional T cell populations were significantly different among early and late stage HPB cancers but at quantitatively much lower frequencies (**Supplementary Figures 4**, **5**).

### Neoadjuvant chemotherapy induced alterations in the T cell compartment and PDA TME niche

**Figure 6:**
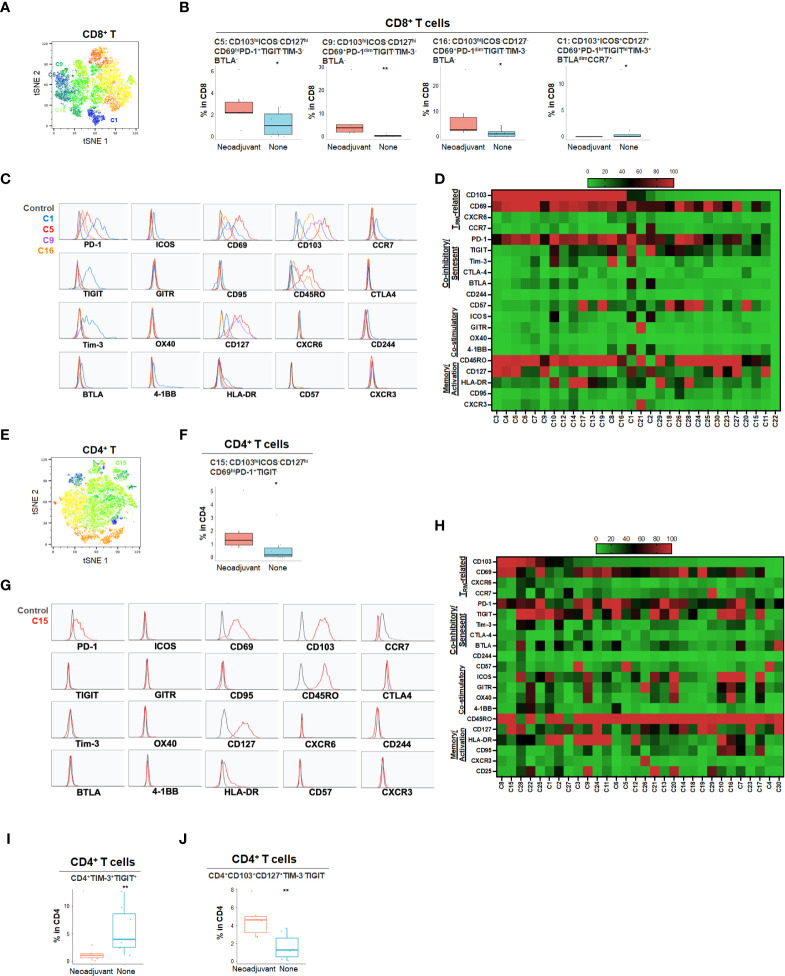
Effect of neoadjuvant chemotherapy on T cell phenotypes in PDA patients **(A)** tSNE projection of CD8+ T cells showing 30 FlowSOM clusters identified from PDA patients treated with (n=6) or without (n=8) neoadjuvant chemotherapy. Significantly enriched clusters in neoadjuvant treatment group (C5, C9, C16) and no treatment group (C1) are labeled. **(B)** Boxplots showing phenotypes of CD8+ T cell clusters enriched following neoadjuvant chemotherapy. Box middle lines, median; box limits upper and lower quartiles; box whiskers, 1.5x the interquartile range. *p<0.05, **p < 0.01, Kruskal-Wallis test. **(C)** Histograms show the single marker phenotypes of CD8+ T cell clusters C5 (red), C9 (purple) and C16 (orange) enriched in PDA patients treated with neoadjuvant chemotherapy and cluster C1 (blue) enriched in PDA patients without neoadjuvant treatment. **(D)** Expression of TRM-related markers, co-inhibitory/senescent markers, co-stimulatory markers and memory/activation markers in 30 clusters of CD8+ T cells is shown in heatmap which is arranged based on the expression level of CD103. **(E)** tSNE projection of CD4+ T cells showing 30 FlowSOM clusters identified from PDA patients treated with (n=7) or without (n=9) neoadjuvant chemotherapy. Significantly enriched cluster C15 in neoajuvant treatment group is labeled. **(F)** Boxplot shows cluster C15 (p<0.05) increased in CD4+ T cells from PDA patients treated with neoadjuvant. **(G)** Histograms show the phenotype of C15 (red) in CD4+ T cells from PDA patients treated with neoadjuvant chemotherapy. **(H)** Expression of TRM-related markers, co-inhibitory/senescent markers, co-stimulatory markers and memory/activation markers in 30 clusters of CD4+ T cells is shown in heatmap which is arranged based on the expression level of CD103. **(I, J)**. Combinatoric frequency analysis of CD4+ marker combinations in response to neoadjuvant chemotherapy (TerraFlow-Methods). Boxplots showing representative phenotypes of CD4+ T cells that are significantly higher in treatment-naïve PDA patients **(I)** or patients receiving neoadjuvant chemotherapy **(J)**.

Neoadjuvant chemotherapy results in a cytotoxic tumor response and has been hypothesized to increase neo-antigen presentation and potentially alter the TME. To characterize the effect of neoadjuvant chemotherapy in PDA patients, we compared patients who had received neoadjuvant chemotherapy (n=7) to those who did not receive chemotherapy (n=9) (**Figure 6** and **Supplementary Figures 6**, **7**). Following treatment, there was no significant change in overall CD4 and CD8 T cell percentage (data not shown), however, there was a significant increase in CD103^hi^CD69^+^ CD8^+^ T_RM_ (C5, C9 and C16) and CD4^+^ T_RM_ (C15) that expressed PD-1 but not TIGIT or Tim-3, suggesting activation of T_RM_ following neoadjuvant chemotherapy (**Figures 6A–H**). However, these T_RM_ expressed PD-1 but lacked co-stimulatory checkpoints (**Figures 6C, D, G, H**), which may render them less effective against tumor cells. On the contrary, we also identified a relatively small frequency population of CD8^+^ T_RM_ (C1) enriched in treatment-naïve PDA patients (**Figure 6B**). Those T_RM_ were triple positive for PD-1, TIGIT and Tim-3, indicating a highly immune inhibitory phenotype (**Figures 6C, D**). Neoadjuvant-treated patients showed a significant reduction of Tim-3^+^TIGIT^+^ CD4^+^ T cells (**Figure 6I**) while there was an enrichment of Tim-3^-^TIGIT^-^CD4^+^ T_RM_ after neoadjuvant treatment (**Figure 6J**), suggesting early T cell activation after neoadjuvant treatment without expansion of a highly exhausted phenotype. Indeed, TIGIT and Tim-3 in CD4^+^ T cells are the most critical set of markers that distinguish naïve versus neoadjuvant-treated groups (**Supplementary Figure 6E**). While the overall populations of PD-1^+^ or TIGIT^+^ T cells remained stable following chemotherapy, the Tim-3^+^ CD8^+^ and CD4^+^ T cells were significantly reduced following treatment, indicating a decrease of an exhausted T cell phenotype (**Supplementary Figure 7E**).

### T cell Phenotypes in PDA primary tumors and liver metastases

We next examined whether there was any alteration in the T cell immune contexture among PDA liver metastatic tumors versus primary PDA tumors. PDA liver metastases demonstrated higher CD8^+^ T_EM_ (**Supplementary Figure 8A–D**) and CD4^+^ T_EM_ (**Supplementary Figures 8E–H**), exhibiting an activated phenotype with CD127 and/or HLA-DR expression. Conversely, PDA primary tumors had greater PD-1^+^ICOS^-^ CD8^+^ T_RM_ (CP5, **Supplementary Figures 8B–D**) and PD-1^hi^TIGIT^+^CD57^+^ senescent CD4^+^ memory T cells (C4, **Supplementary Figures 8F–H**), suggesting a more chronic exhausted and/or senescent state in the primary tumor compared to liver metastasis. (**Supplementary Figure 8C, D, G–J**). Interestingly, we also found a small population of T_RM_ enriched in primary PDA expressing both co-stimulatory, ICOS and 41-BB, and co-inhibitory, PD-1, TIGIT, Tim-3 and BTLA, immune checkpoint receptors, as well as CD95 (Fas) that leads to apoptotic cell death (C9, **Supplementary Figures 8I, J**). This T_RM_ population may represent activated T cells with an exhausted phenotype and may be prone to activation-induced apoptosis.

## Discussion

In this study, we utilized high-parameter flow cytometry for the first time to characterize the phenotypic and functional state of tumor infiltrating T cell populations across HPB malignancies. We conducted a broad and comparative assessment of immune checkpoint receptors, trafficking receptors, activation and phenotype markers at the protein level and identified key differences in T cell states among HCC, PDA and CCA that mapped to clinical features of these diseases. This novel technology has multiple potential advantages over mass cytometry (CyTOF), such as increased sensitivity and detection of T cells in scant tumor samples including core needle biopsies. Furthermore, in comparion to other large HCC cohorts utilizing RNA sequencing and/or immunohistochemistry, our study is largely consistent with the previous studies, with our study having the added benefit of simultaneously measuring multiple cell surface markers concurrently to better define immune cell populations (23, 24). This was a particular advantage in our current study in that we were able to assess patients with advanced stages of disease where tissue is only obtainable with core needle biopsy. Thus we were able to directly 1) compare T cell subsets in HPB malignancies, 2) identify whether clinical prognosis correlates with T cell populations, and 3) for PDA patients, determine the effects of chemotherapy and site of disease on tumor T cell populations. Additionally, we were specifically able to characterize across these disease states the presence of T cell populations that may be targetable using immunological modifying drugs in clinical development.

Although protein analysis using our unique high parameter flow cytometry approach offers a powerful tool for characterizing TIL, there are limitations to this study. First, we did not perform direct measurements of cell function; thus, we cannot confirm that cells expressing immune checkpoints commonly classified as coinhibitory are truly anergic or inhibited *in vivo*. Second, we did not report expression for the ligands of the immune checkpoint molecules we analyzed; engagement of these ligands is typically required for eliciting inhibitory or activating pathways in T-cells. Third, it is unknown how many co-inhibitory or co-stimulatory molecules are required to engage an activating or inhibiting pathway in a T-cell. It may be that expression levels below the limit of detection of our flow cytometry assays are sufficient to change a cellular program.

Most tumor-infiltrating T cells we examined were of an antigen-experienced CD45RO^+^ memory phenotype. Memory T cells comprise diverse subsets including central memory (T_CM_), effector memory (T_EM_) and tissue-resident (T_RM_) memory T cells. Their generation and functional state are dynamically shaped by tissue site and tumor microenvironment (6). Single marker evaluation highlighted the similar presence of CD45RO^+^ memory T cells and CD103^+^ T_RM_, while T_CM_ trended higher in HCC patients. CXCR6^+^ T cells and CD69^+^ CD4^+^ T cells were also significantly higher in HCC patients, suggesting better T cell trafficking and retention in HCC tumors compared to PDA. In addition, CD57^+^ terminally differentiated effector T cells were present in the three cancer types, with a slight increase of CD57^+^CD4^+^ T cells in HCC compared to PDA. Furthermore, we profiled both co-stimulatory and co-inhibitory immune checkpoint receptors. Co-inhibitory checkpoint receptors were generally more prevalent than co-stimulatory checkpoint receptors, with PD-1 and TIGIT being the most frequently expressed followed by TIM3 and BTLA in HPB cancer patients. CTLA4 was among the lowest frequencies of checkpoint receptors in HPB cancer patients. PD-1 was noted on over 46% and 35% of CD8^+^ and CD4^+^ T cells in HCC patients, respectively, but was much more heterogenous in PDA (5%-85% for CD8^+^ and 3%-75% for CD4^+^) and CCA (24%-80% for CD8^+^ and 10%-76% for CD4^+^) patients. Notably, ICOS was expressed at higher levels in both liver cancers than in PDA, suggesting tumor-tissue specific features and a dysfunctional T cell state in PDA. While single marker analysis was notable, we sought to further define the unique T cell populations and states across these HPB diseases and match our results to clinical parameters.

Therefore we leveraged the power of multiparameter flow cytometry to explore the diverse phenotypic and functional states of T cell subsets. Utilizing the bioinformatics techniques of dimension reduction and clustering *via* tSNE and the combinatoric TerraFlow platform, unbiased analysis of multiple simultaneous T cell surface markers was possible, as has been recently described for peripheral T cells in advanced melanoma patients (10). In PDA we noted extremely small populations of partially exhausted T cells, most of them consistent with terminally exhausted T cells expressing co-inhibitory but not co-stimulatory immune checkpoint receptors. These findings may explain the relative lack of response rates to anti-PD-1 therapy in PDA (25, 26). In HCC patients, generally known for better anti-PD-1 treatment response rates of 15-20% (4, 27), we found a higher frequency of partially exhausted T_RM_ cells, expressing not only elevated levels of PD-1, but also TIGIT, Tim-3, and the co-stimulatory checkpoint ICOS. T_RM_ is positively associated with response to ICI in several cancer types. In cancer patients responding to anti-PD-1 or anti-PD-1/CTLA4, clonally expanded T cells showed elevated T_RM_ and cytotoxicity programs, suggesting rapid response capability of T_RM_ in response to ICI (28). This suggests that targeting of PD-1 or its ligand in HCC can occasionally be successful, and resistance to anti-PD-1/PD-1 ligand monotherapy in HCC may be overcome by combination of agents directed simultaneously against these co-inhibitory and co-stimulatory checkpoint signals. Interestingly, this partially exhausted T cell phenotype (T_PEX_) was more commonly noted in early stage HCC. This provides rationale to consider multimodality therapies for earlier stage HCC and that over time, a more terminally exhausted T cell state develops in more advanced disease. In contrast to HCC, advanced stage PDA patients had a higher frequency of naïve T cells, suggesting that increased antigen turnover and presentation may be required for success of immunotherapy in PDA. Early stage PDA patients contained higher activated T_RM_ cells with TIGIT or PD-1 than late stage PDA patients, but were lacking co-stimulatory molecules and altogether were much lower in frequency than in HCC patients. This indicates early stage PDA harbored terminally exhausted T cells, partially explaining the failed response of PD-1 immunotherapy in PDA. Further, we were able to quantify the presence and level of expression of checkpoint inhibitors and activators, including their functional status, function, i.e. exhaustion status, in T cell subsets in a comprehensive, unbiased manner. There is limited data about checkpoint molecule co-expression of T cells in HCC, PDA, and CCA and their clinical relevance.

It has been shown that in the absence of co-stimulation, the tumor-antigen specific TCR signal alone induces T cell anergy rather than activation (29). It has been postulated that cytotoxic chemotherapy might enhance T cell activation by increasing antigen turnover within the TME (30). Comparing samples of PDA patients treated with neoadjuvant chemotherapy with treatment naïve samples, we observed a statistically significant increase in activated CD8^+^ and CD4^+^ T_RM_ cells with treatment, characterized by high CD103, CD127, CD69, and PD-1 expression. The clinical significance of this change is unclear given the relative paucity of other checkpoint receptors and the small size of these T cell populations (less than 2-3% of T cells in all patients). Similarly, there was a reduction from the baseline percentage of CD4^+^Tim-3^+^TIGIT^+^ of exhausted T cells but an induction of CD4^+^Tim-3^-^TIGIT^-^CD127^+^ T_RM_ following neoadjuvant treatment. A recent study showed a relative increase in PD-1, TIGIT, and 4-1BB expression on peripheral circulating T cells (outside of the TME) in PDA patients receiving cytotoxic chemotherapy (31), but did not assess for other T cell checkpoint receptors or activation markers or intra-tumoral T cells. Other than these changes in isolated, small populations of T cells, there was no evidence of significant induction of either 1) increased T cell trafficking and differentiation, 2) increased T cell activation, or 3) robust checkpoint molecule expression changes in PDA patients following chemotherapy. When comparing primary PDA samples to PDA liver metastases, we noticed higher T cell activation but lack of a core T_RM_ phenotype in metastatic samples, suggesting organ specific differences in TME complexity and consequently potential differential susceptibility to immunotherapies.

One strength of our study is the ability to study checkpoint molecule co-expression and other T cell marker surface protein expression and assess its clinical/translational relevance by quantification of checkpoint molecule expression and other markers in relation to each other, the disease stage or the exhaustion profile. Similar to the previous studies investigating the TME in human HCC using a variety of complex immunophenotyping techniques (32–34), our study not only assessed similar broad parameters, but also served to extend the detail of the phenotypic analysis of these T cell subsets, particularly across disease states, stage, and in the case of PDA, in the context of chemotherapy. The information gained by these analyses aids in understanding which T cell populations are present in patient tumors to help guide future decision making regarding checkpoint targeting therapy. A weakness of our study is that while our platform allowed for simultaneous assessment of 25 markers on small portions of tissue, additional platforms (e.g. intracellular markers and myeloid subsets) could not be simultaneously assessed due to finite amounts of tissue. It is also possible that varying chemotherapy regimens may have differing effects on the T cell subsets in the TME of PDA patients and similarly differing metastatic sites may possess a varying TME in PDA patients.

In summary, we have identified unique T cell states in the TME of hepatobiliary and pancreatic malignancies. We further identified altered T cells states related to disease stage in HCC and PDA. Terminally exhausted T cells were more prevalent in a graded fashion from HCC, to CCA, and ultimately PDA, whereas functionally exhausted T cells, capable of restored activation, were most prevalent in HCC, especially in early stage HCC. With data from preclinical studies of HCC showing that combined therapy targeting multiple inhibitory checkpoint receptors exhibited synergistic treatment efficacy by restoring TILs-mediated anti-tumor immunity (35, 36) and the detailed immunophenotyping presented in this study, HCC patients may benefit most from combined checkpoint therapies whereas efforts to increase overall T cell activation in CCA and PDA warrant further investigation.

## Data availability statement

The original contributions presented in the study are included in the article/**Supplementary Material**. Further inquiries can be directed to the corresponding author.

## Ethics statement

The studies involving human participants were reviewed and approved by Perlmutter Cancer Center, NYU Langone Health. The patients/participants provided their written informed consent to participate in this study.

## Author contributions

SW, EZ, PC, DiS and TW developed the study concept and were responsible for the study design. SW, EZ, DW, DiS. and TW wrote the manuscript, which was then revised and approved by all co-authors. SW, EZ, GW and DF helped acquire human biospecimens and processed samples for flow analysis. SW, EZ and PC analyzed the flow data. SW and DF were responsible for the R and combinatorial analysis. SW, EZ, DW, BK, GW, LK, DeS, and PO collected patient information and maintained the databases. BK, LK, DeS, PO and PC provided study guidance and feedback on the manuscript. PC, DiS and TW directed the study. DiS and TW provided grant support. All authors contributed to the article and approved the submitted version.
