# Corrigendum: Tumor infiltrating T cell states and checkpoint inhibitor expression in hepatic and pancreatic malignancies

**DOI:** 10.3389/fimmu.2023.1177713

**Published:** 2023-03-23

**Authors:** Shanshan Wan, Ende Zhao, Daniel Freeman, Daniel Weissinger, Benjamin A. Krantz, Gregor Werba, Lauren G. Khanna, Despina Siolas, Paul E. Oberstein, Pratip K. Chattopadhyay, Diane M. Simeone, Theodore H. Welling

**Affiliations:** ^1^ Department of Surgery, NYU Langone Health, New York, NY, United States; ^2^ Perlmutter Cancer Center, NYU Langone Health, New York, NY, United States; ^3^ Pathology, NYU Langone Health, New York, NY, United States; ^4^ Internal Medicine, NYU Langone Health, New York, NY, United States; ^5^ Talon Biomarkers, Mendham, NJ, United States

**Keywords:** pancreatic adenocarcinoma, hepatocellular carcinoma, cholangiocarcinoma, immune oncology, tumor immunity, T cell biology, checkpoint inhibitors

In the published article, there was an error regarding the affiliation for Pratip K. Chattopadhyay.As well as having affiliations 2,4, they should also have Talon Biomarkers, Mendham, NJ, United States.

In the published article, there was an error in the author list, and author Daniel Freeman was erroneously listed as 6^th^ author. Author Pratip K. Chattopadhyay was erroneously not listed as a co-corresponding author. Author Diane M. Simeone was erroneously not listed as a co-corresponding author. The corrected author list appears below.

Shanshan Wan^1†^, Ende Zhao^1,2†^, Daniel Freeman^4^, Daniel Weissinger^2^, Benjamin A. Krantz^2,3^, Gregor Werba^2^, Lauren G. Khanna^3^, Despina Siolas^2,3^, Paul E. Oberstein^2,3^, Pratip K. Chattopadhyay^2,4,5*^, Diane M. Simeone^1,2,4*^, Theodore H. Welling^1,2*^

In the published article Freeman D, Lam L, Le T, Alexandre J, Raphael B, Grossbard M, et al. TerraFlow, A New High Parameter Data Analysis Tool, Reveals Systemic T-cell Exhaustion and Dysfunctional Cytokine Production in Classical Hodgkin Lymphoma. *medRxiv.* 2022 was not cited in the article. The citation has now been inserted in **Methods**, *Combinatorial Analysis* and should read:

“CD4^+^ and CD8^+^ T cell data were exported respectively for each sample for rapid computation of combinatorial phenotypes using the CytoBrute platform (RocketML) (10). Phenotypes consisting of every single marker, and all possible combinations of 2-15 markers were constructed, and enumerated across all samples. The top 1,000 most frequent combinatorial phenotypes were reported and compared across study groups. For some analyses, flow cytometry data files, gating thresholds and comparison groups were uploaded to TerraFlow (TerraFlow.app) for construction of all possible 1-5 marker phenotypes across the dataset. Phenotypes whose frequency differed statistically significantly between phenotypes were reported. TerraFlow also generated core phenotypes that summarize the most significant families of phenotypes. Finally, a simple set of markers that distinguished groups was identified using recursive feature elimination (13).”

In the published article, there was an error in the Funding statement. Funding from one author (PKC) was missing. The correct Funding statement appears below.

**FUNDING**

“This study was funded by NIH R01CA245005 (DMS), P30CA016087 and Perlmutter Cancer Center Developmental Projects Program (CCSG) (THW) and NYULH research program startup funds (PKC).”

In the published article, there was an error in the Conflict of Interest. The Conflict of Interest was not correctly disclosed. This sentence previously stated: “The authors declare that the research was conducted in the absence of any commercial or financial relationships that could be construed as a potential conflict of interest.” The correct Conflict of Interest appears below.

**Conflict of Interest**

“DF and PC own TerraFlow, which was used for some of the analyses presented in the paper.

The remaining authors declare that the research was conducted in the absence of any commercial or financial relationships that could be construed as a potential conflict of interest.”

In the published article, there was an error. missing or misleading texts.

A correction has been made to **Abstract**. This sentence previously stated:

“We therefore defined T cell subsets in the tumor microenvironment of HPB patients utilizing a novel, multiparameter flow cytometry and bioinformatics analysis. Our findings quantify the T cell phenotypic states in relation to checkpoint receptor expression. We demonstrate the presence of CD103^+^ tissue resident memory T cells (T_RM_), CCR7^+^ central memory T cells, and CD57^+^ terminally differentiated effector cells across all HPB cancers, while the anti-tumor function was dampened by expression of multiple co-inhibitory checkpoint receptors. Terminally exhausted T cells lacking co-stimulatory receptors were more prevalent in PDA, whereas partially exhausted T cells expressing both co-inhibitory and co-stimulatory receptors were most prevalent in HCC, especially in early stage. HCC patients had significantly higher T_RM_ with a phenotype that could confer restored activation in response to immune checkpoint therapies. Further, we found a lack of robust alteration in T cell activation state or checkpoint expression in response to chemotherapy in PDA patients.”

The corrected sentence appears below:

“We therefore defined T cell phenotypic states, particularly in terms of immune checkpoint receptor expression, in the tumor microenvironment of HPB patients utilizing novel, multiparameter flow cytometry and bioinformatics analysis. We demonstrate the presence of CD103^+^ tissue resident memory T cells (T_RM_), CCR7^+^ central memory T cells, and CD57^+^ terminally differentiated effector cells across all HPB cancers, with simultaneous expression of multiple co-inhibitory checkpoint receptors. Terminally differentiated T cells lacking co-stimulatory receptors were more prevalent in PDA, whereas T cells expressing both co-inhibitory and co-stimulatory receptors were most prevalent in HCC, especially in early stage. HCC patients had significantly higher T_RM_ with a phenotype that might confer restored activation in response to immune checkpoint therapies. Further, T-cell activation state and checkpoint expression did not change robustly in response to chemotherapy in PDA patients.”

In the published article, there was an error. missing or misleading texts.

A correction has been made to **Methods**, *High Parameter Flow Cytometry*. This sentence previously stated:

“A 25 parameter antibody panel was developed using ColorWheel software (10) to include T cell markers of differentiation, activation, trafficking, phenotype, co-inhibitory and co-stimulatory checkpoint molecules CD186 (B515), CD137 (B610), CD244 (B710), CD57 (B780), CD45RO (R670), HLA-DR (R710), GITR (R780), CD278 (V420), CD95 (V475), CD103 (V610), CD183 (V670), CD134 (V710), CD69 (V740), CD4 (V780), CCR7 (U380), Viability dye (U450), CD3 (U515), CD25 (U585), CD366 (U670), PD-1 (U740), CD8 (U820), TIGIT (G575), CD272 (G610), CD127 (G670) and CD152 (G780) (BD Biosciences)(10). Data were collected on a custom BD FACSymphony A5 30-parameter Cell Analyzer and compensated and analyzed with FlowJo software (BD Biosciences). FACS data were checked for quality of staining and normalized. Manual compensation was done for overlap in fluorescent spectra and fluorescence aggregates, dead cells and cell doublets were excluded. Fluorescence intensity thresholds and gates were then determined for each marker to distinguish positive from negative expression. Data from CD4^+^ and CD8^+^ T cells were analyzed separately.”

The corrected sentence appears below:

“A 25 color antibody panel was developed using ColorWheel software (10) to include T cell markers of differentiation, activation and trafficking along with co-inhibitory and co-stimulatory checkpoint molecules. The following mouse anti-human antibodies were used, all purchased from BD Biosciences, except where indicated: CD186 (Brilliant Blue (BB)515), CD137 (BB630), CD244 (BB700), CD57 (BB780), CD45RO (allophycocyanin (APC)), HLA-DR (R700APC), GITR (Cyanin 7 (Cy7)-APC), CD278 (Brilliant Violet (BV)421)), CD95 (BV510), CD103 (BV605), CD183 (BV650), CD134 (BV705), CD69 (BV750), CD4 (BV785), CCR7 (Brilliant Ultraviolet (BUV) 395), LIVE/DEAD Fixable Blue (ThermoFisher Scientific, Carlsbad, CA), CD3 (BUV496), CD25 (BUV563), CD366 (BUV661), PD-1 (BUV737), CD8 (BUV805), TIGIT (phycoerythrin (PE)), CD272 (PE-CF594), CD127 (PE-Cy5) and CD152 (PE-Cy7) (10). Data were collected on a custom BD Biosciences FACSymphony A5 30-parameter flow cytometer, then compensated and analyzed with FlowJo software (BD Biosciences). Data were checked for quality of staining and fluorescence aggregates, dead cells and cell doublets were excluded. Fluorescence intensity thresholds were then determined for each marker to distinguish positive from negative expression.”

In the published article, there was an error. missing texts.

A correction has been made to **Results**, *T cell Distribution in HPB Cancers*, *Paragraph 1*. This sentence previously stated:

“T_CM_ in HCC trended higher when compared to T_CM_ in PDA and CCA, but did not reach statistical significance, based on CCR7 as a single marker (**Figures 1B, C**).”

The corrected sentence appears below:

“T_CM_ in HCC trended higher when compared to T_CM_ in PDA and CCA, but did not reach statistical significance, based on CCR7 as a single marker (**Figures 1B, C**). CD127 (the IL7-receptor) also commonly marks TCM, but exhibited a different pattern than CCR7, as it trended to higher levels (amongst CD8^+^ T-cells) or is significantly elevated (amongst CD4^+^ T-cells) in PDA and CCA compared to HCC.”

In the published article, there was an error. missing or misleading texts.

A correction has been made to **Results**, *T cell phenotypes unique to HPB cancer type*, *Paragraph 1*. This sentence previously stated:

“High parameter flow cytometry for T cell subtype analysis allows for the possibility of 10^10^ theoretical phenotypes of T cells based on the possible combinations of cell surface marker expression.”

The corrected sentence appears below:

“Using specialized data analysis tools, like CytoBrute and TerraFlow, high parameter flow cytometry data can be mined to examine all the theoretically possible phenotypes. In this study, as many as 3^22^ theoretical phenotypes may be present in the data (where three conditions – positive, negative, and omitted are analyzed for each of 22 markers). Because some markers were rarely expressed, and because of the challenge of interpreting long higher-order phenotypes of marker combinations, we limited CytoBrute analysis to 15 markers and TerraFlow analysis was designed to sample all possible sets of six markers. Cell phenotypes that were exceedingly rare were removed from downstream analysis and comparison across patient groups.”

In the published article, there was an error. missing or misleading texts.

A correction has been made to **Results**, *T cell phenotypes unique to HPB cancer type*, *Paragraph 1*. This sentence previously stated:

“This observation was independently validated by combinatoric analysis using the TerraFlow platform (**Figure 2E**).”

The corrected sentence appears below:

“We used TerraFlow to provide further insight into the results observed in our other analyses. TerraFlow identified a number of phenotypes, not revealed by tSNE analysis, that differed between HCC and PDA (**Figure 2E**), variously highlighting the elevation of ICOS, TIGIT, CD69, and PD1 in distinguishing PDA from HCC (Supplemental Figures 2A–E) using machine learning and recursive feature analysis(13). TerraFlow revealed that HCC was highly enriched for ICOS+ TRM that frequently expressed PD-1 and/or TIGIT (**Figure 2E**), suggesting that immune checkpoint blockade of PD1 and/or TIGIT might release inhibition of the already-expressed costimulatory molecule ICOS. Supplemental Figures 2C and F illustrate the p-values for all phenotypes compared between HCC and PDA.”

In the published article, there was an error. missing or misleading texts.

A correction has been made to **Results**, *T cell phenotypes unique to HPB cancer type*, *Paragraph 2*. This sentence previously stated:

“All of these PDA-enriched T cell populations shared a striking characteristic which is the absence of all four co-stimulatory immune checkpoint receptors, ICOS, GITR, OX40 and 4-1BB (**Figures 3D–G**, Supplemental Figure 3F), suggesting T cell anergy. These observations were independently validated by combinatoric TerraFlow platform (**Figures 2H**, **3H**). These findings indicate the tumor microenvironment in PDA could suppress activation of anti-tumor T cells and/or induce T cell anergy *via* repressing co-stimulatory immune checkpoint signals while maintaining co-inhibitory immune checkpoint signals”

The corrected sentence appears below:

“All of these PDA-enriched T cell populations shared a striking characteristic which is the absence of all four co-stimulatory immune checkpoint receptors, ICOS, GITR, OX40 and 4-1BB (**Figures 2H**, **3D–H**, Supplemental Figure 3F), suggesting T cell anergy. These findings suggest the tumor microenvironment in PDA could suppress activation of anti-tumor T cells and/or induce T cell anergy by repressing co-stimulatory immune checkpoint signals”

In the published article, there was an error. missing paragraph 2.

A correction has been made to **Discussion**, *Paragraph 2*. This sentence previously stated:

“Additionally, we were specifically able to characterize across these disease states the presence of T cell populations that may be targetable using immunological modifying drugs in clinical development.”

The corrected sentence appears below:

“Additionally, we were specifically able to characterize across these disease states the presence of T cell populations that may be targetable using immunological modifying drugs in clinical development.

Although protein analysis using our unique high parameter flow cytometry approach offers a powerful tool for characterizing TIL, there are limitations to this study. First, we did not perform direct measurements of cell function; thus, we cannot confirm that cells expressing immune checkpoints commonly classified as co-inhibitory are truly anergic or inhibited *in vivo*. Second, we did not report expression for the ligands of the immune checkpoint molecules we analyzed; engagement of these ligands is typically required for eliciting inhibitory or activating pathways in T-cells. Third, it is unknown how many co-inhibitory or co-stimulatory molecules are required to engage an activating or inhibiting pathway in a T-cell. It may be that expression levels below the limit of detection of our flow cytometry assays are sufficient to change a cellular program”.

The authors apologize for these errors and state that this does not change the scientific conclusions of the article in any way. The original article has been updated.

