# Supplementary figures and images for: Tumor infiltrating T cell states and checkpoint inhibitor expression in hepatic and pancreatic malignancies
Source: Front Immunol. 2023 Jan 31;14:1067352. doi: 10.3389/fimmu.2023.1067352 (PMC9927010; doi:10.3389/fimmu.2023.1067352)

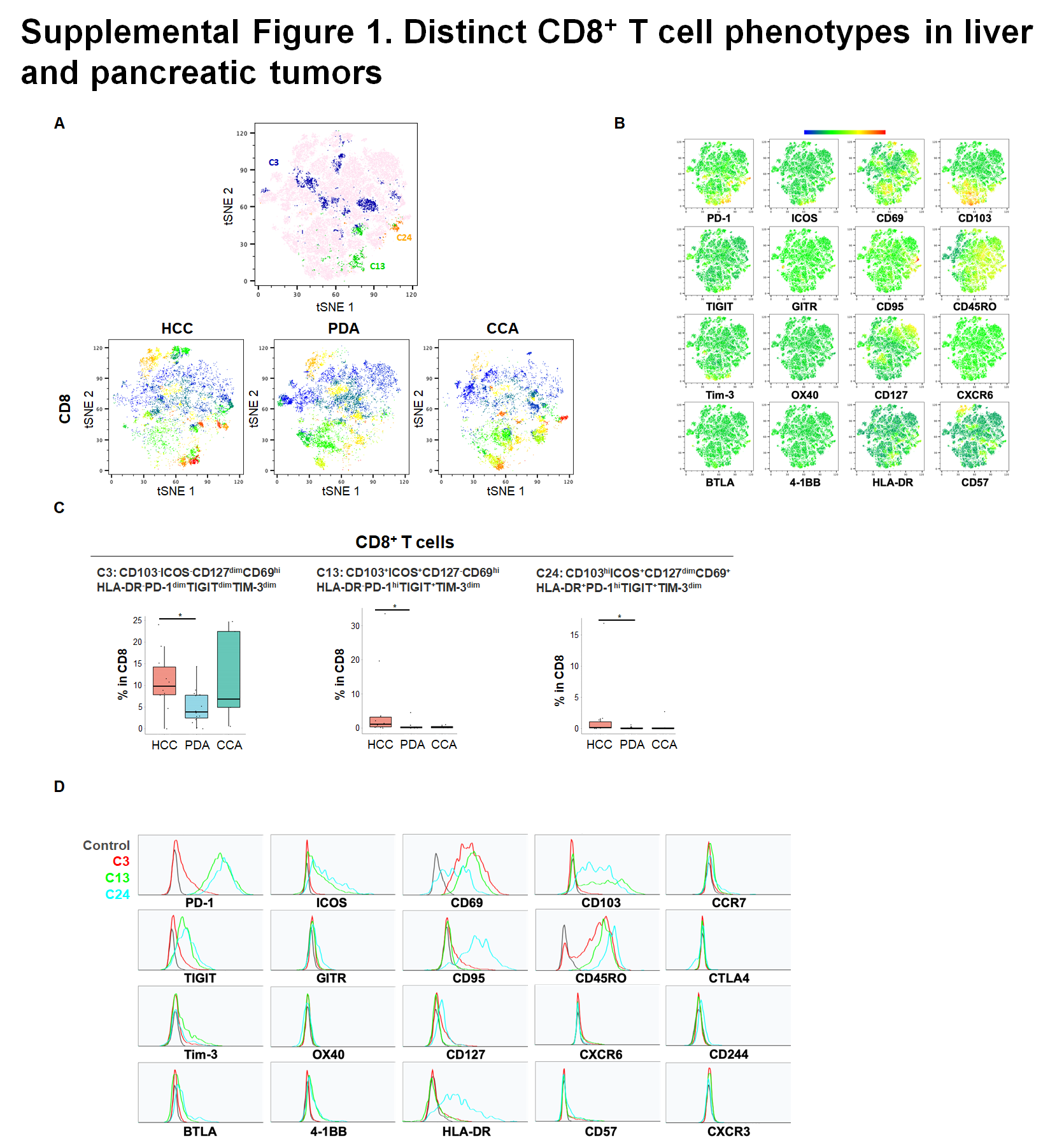

Supplement: Supplementary file 1 [file Image_1.tif]

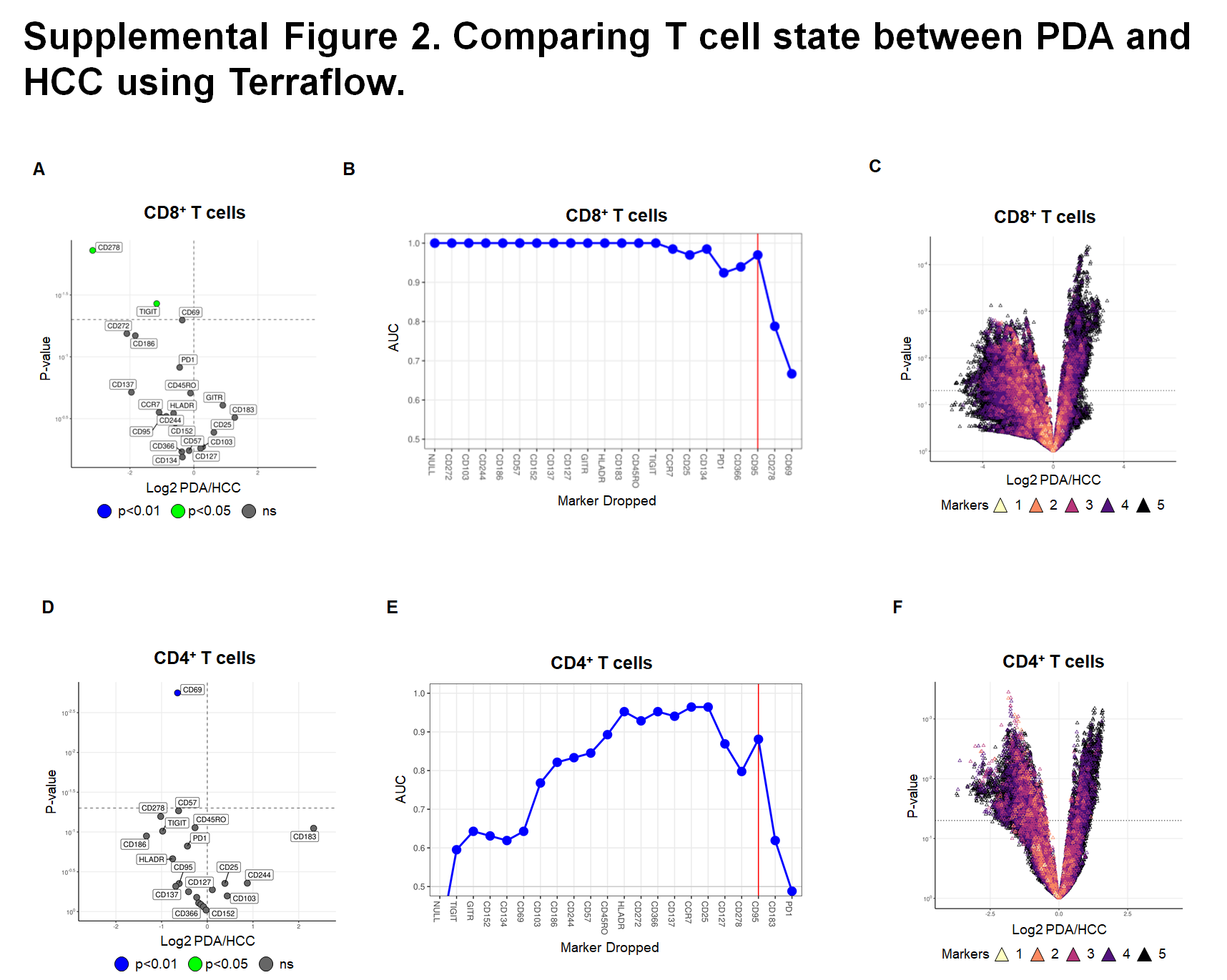

Supplement: Supplementary file 2 [file Image_2.tif]

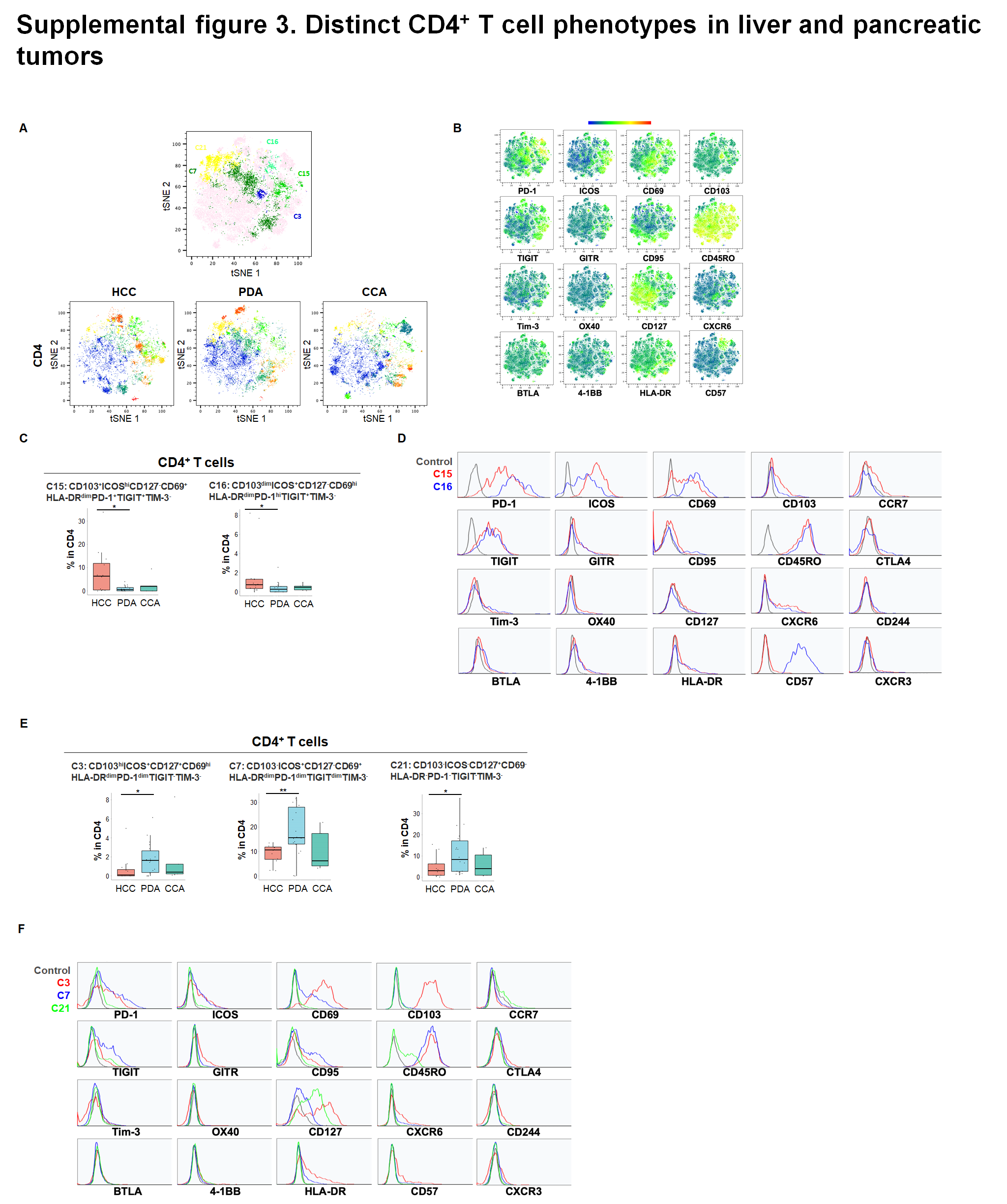

Supplement: Supplementary file 3 [file Image_3.tif]

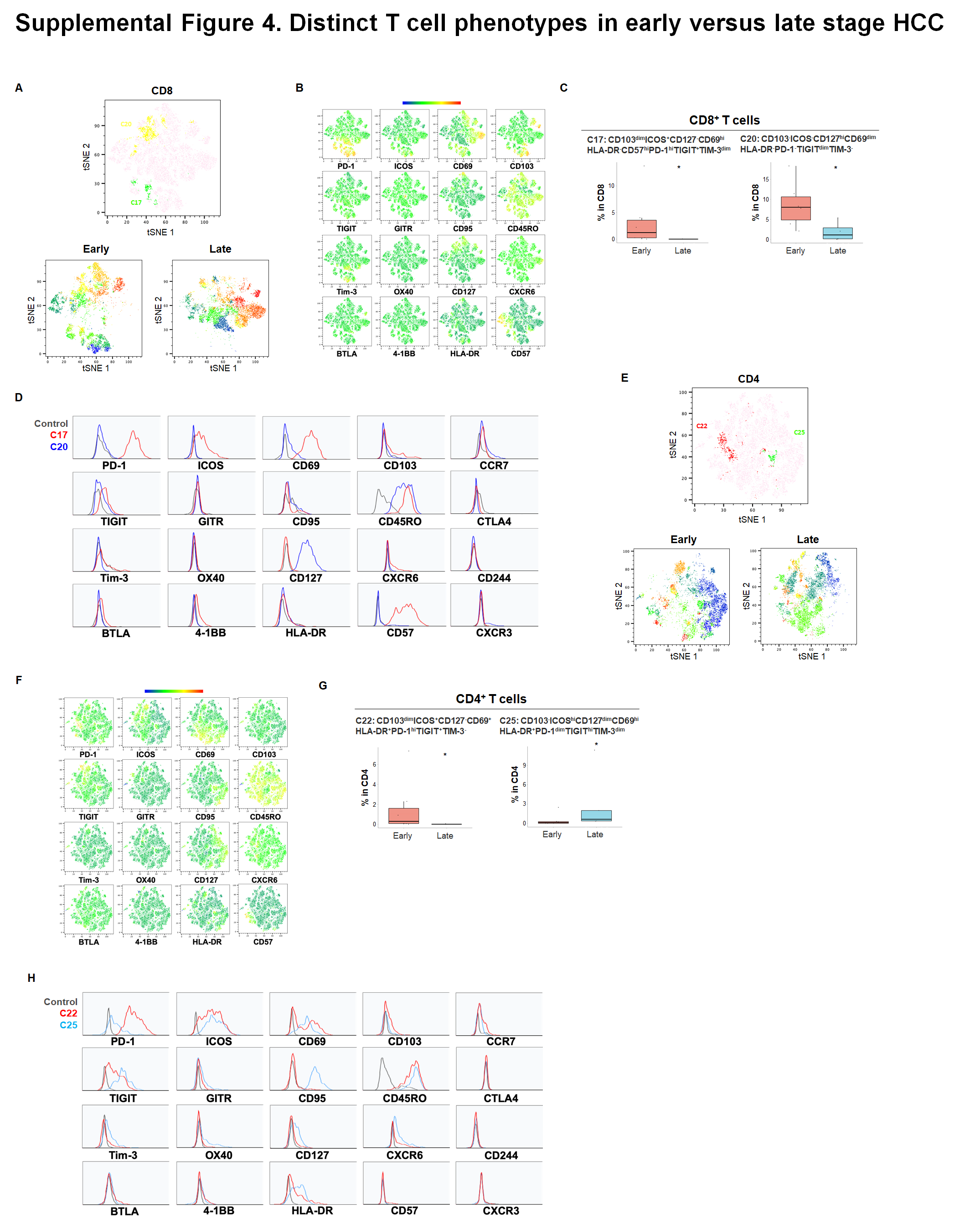

Supplement: Supplementary file 4 [file Image_4.tif]

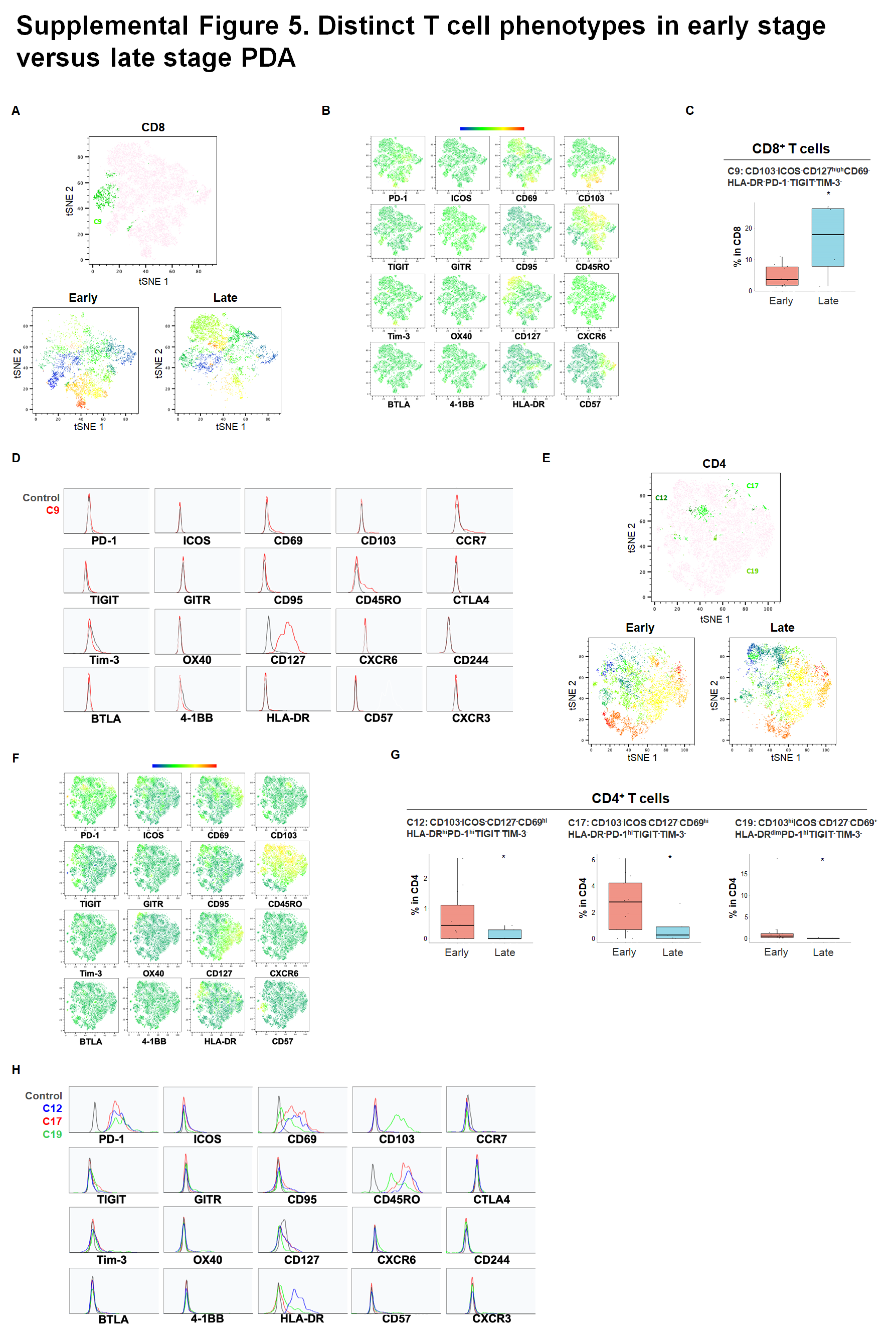

Supplement: Supplementary file 5 [file Image_5.tif]

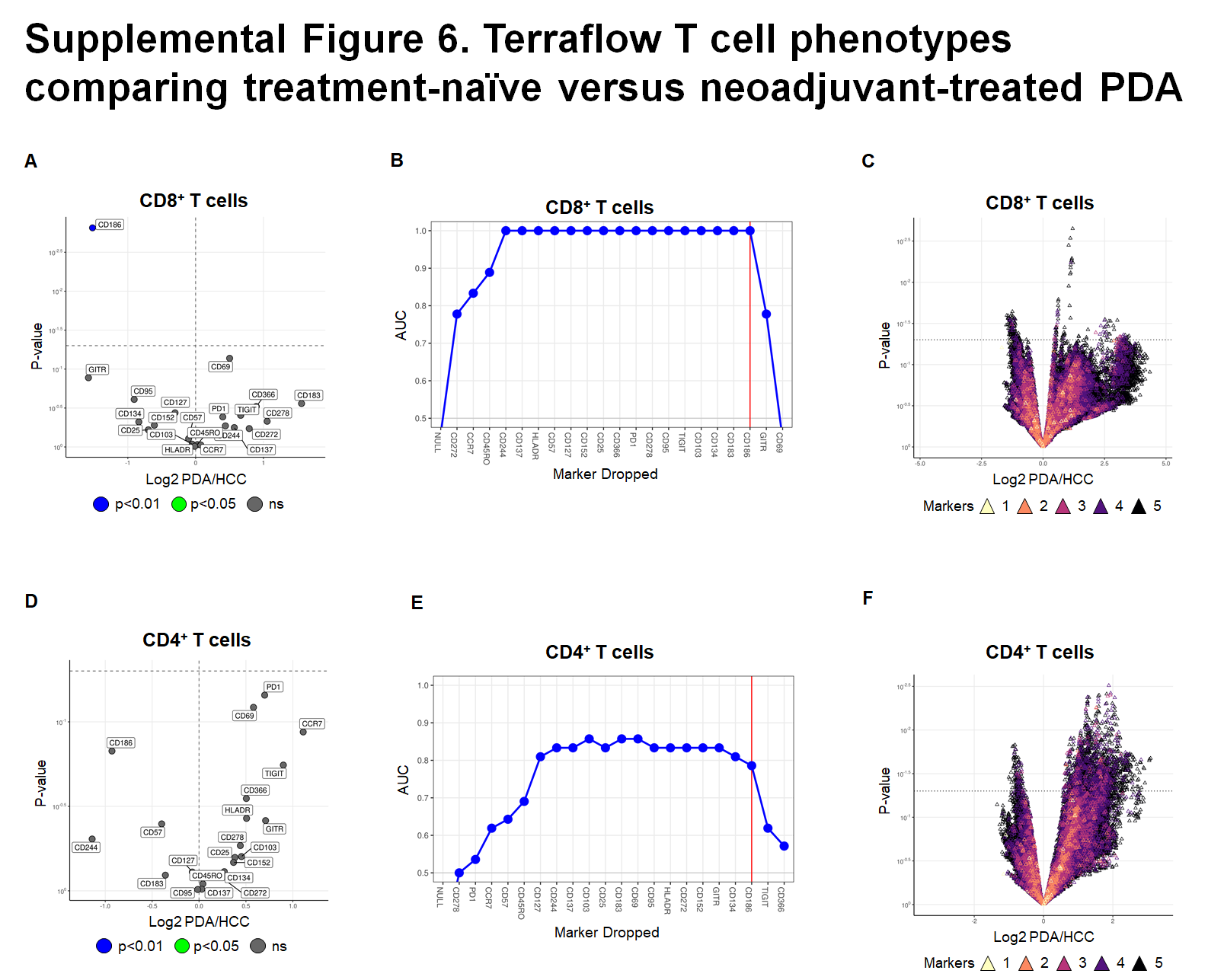

Supplement: Supplementary file 6 [file Image_6.tif]

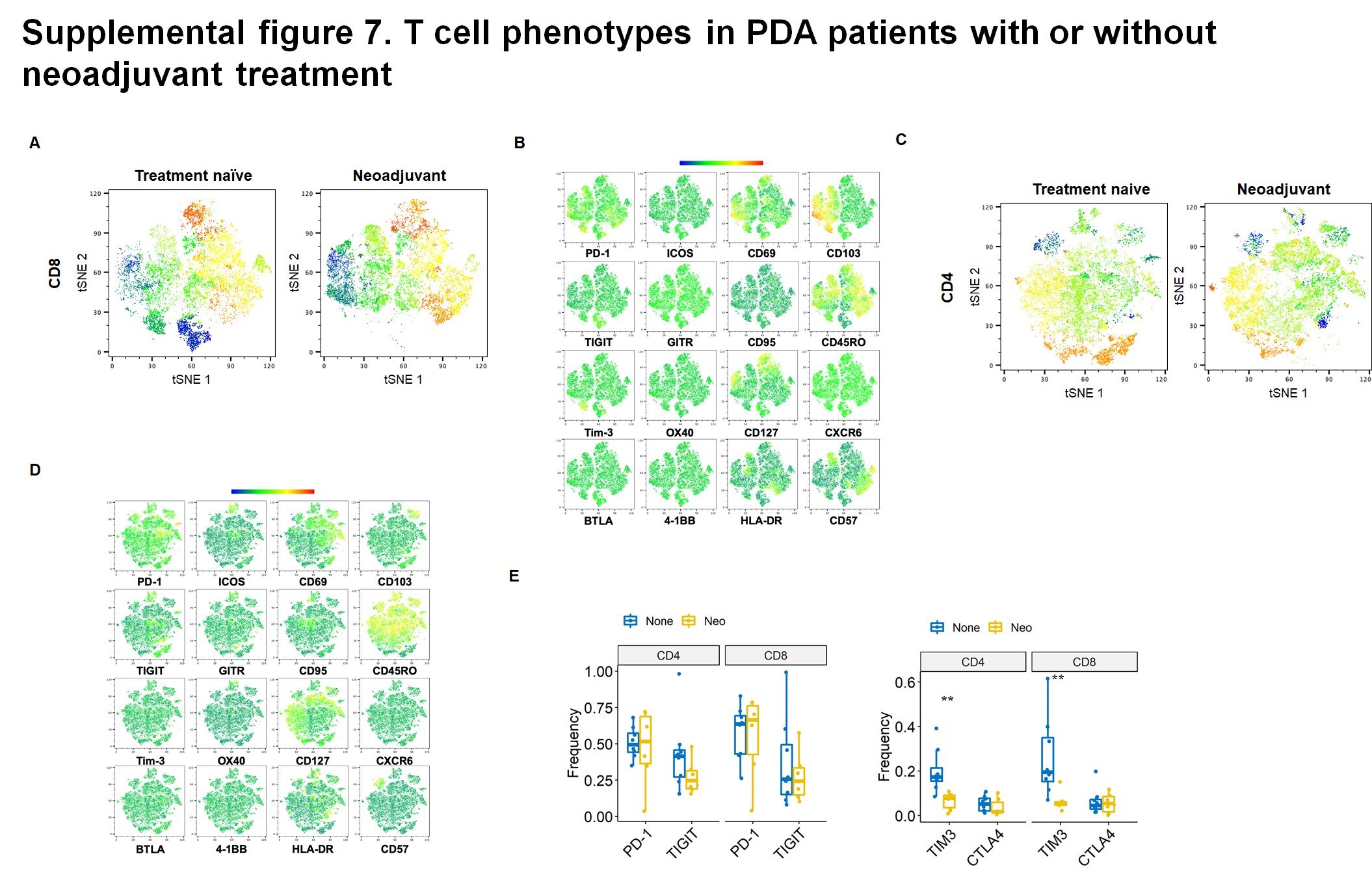

Supplement: Supplementary file 7 [file Image_7.tif]

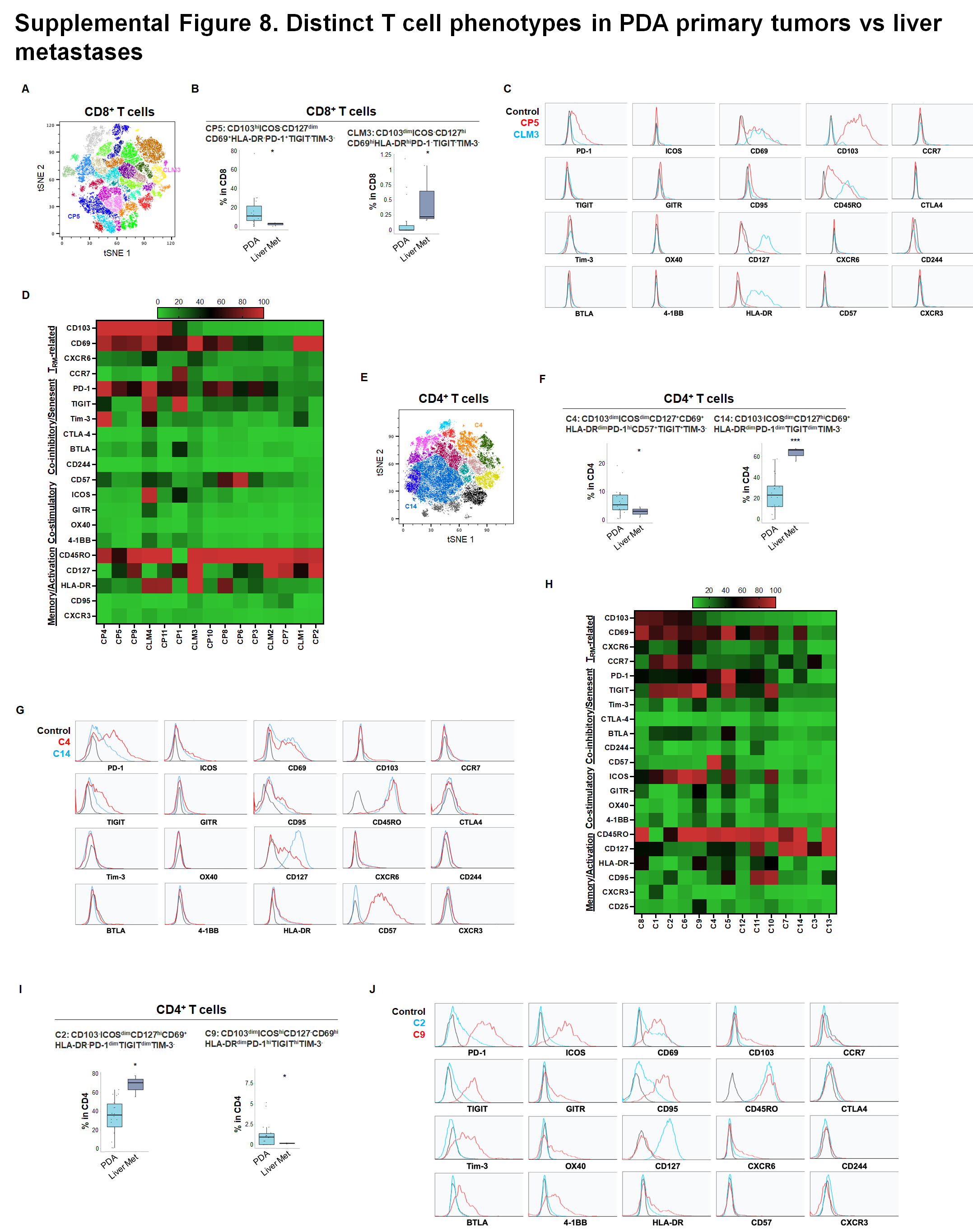

Supplement: Supplementary file 8 [file Image_8.tif]
